# A Narrative Review Discussing the Obstetric Repercussions Due to Alterations of Personalized Bacterial Sites Developed within the Vagina, Cervix, and Endometrium

**DOI:** 10.3390/jcm12155069

**Published:** 2023-08-01

**Authors:** Bogdan Doroftei, Ovidiu-Dumitru Ilie, Theodora Armeanu, Irina-Liviana Stoian, Nicoleta Anton, Ramona-Geanina Babici, Ciprian Ilea

**Affiliations:** 1 Faculty of Medicine, University of Medicine and Pharmacy “Grigore T. Popa”, University Street No. 16, 700115 Iasi, Romania; 2 Clinical Hospital of Obstetrics and Gynecology “Cuza Voda”, Cuza Voda Street No. 34, 700038 Iasi, Romania; 3 Origyn Fertility Center, Palace Street No. 3C, 700032 Iasi, Romania; 4 Department of Genetics, University of Medicine and Pharmacy “Grigore T. Popa”, University Street No. 16, 700115 Iasi, Romania

**Keywords:** microbiota, vagina, cervix, endometrium, early miscarriage, late miscarriage

## Abstract

Background: The reproductive tract microbiota that evolved as an integrative component has been studied intensively in the last decade. As a result, novel research, clinical opportunities, and perspectives have been derived following the close investigation of this microecological environment. This has paved the way for an update to and improvement of the management strategies and therapeutic approaches. However, obscurities, contradictions, and controversies arise regarding the ascension route from the vagina to the endometrium via the cervix, with finality in adverse obstetric outcomes. Methods: Starting from these considerations, we aimed to gather all existing data and information from four major academic databases (PubMed, ISI Web of Knowledge, Scopus, and ScienceDirect) published in the last 13 years (2010–2023) using a controlled vocabulary and dedicated terminology to enhance the coverage, identification, and sorting of potentially eligible studies. Results: Despite the high number of returned entries (n = 804), only a slight percentage (2.73%) of all manuscripts were deemed eligible following two rounds of evaluation. Cumulatively, a low level of *Lactobacillus* spp. and of other core microbiota members is mandatory, with a possible eubiosis-to-dysbiosis transition leading to an impairment of metabolic and endocrine network homeostasis. This transposes into a change in the pro-inflammatory landscape and activation of signaling pathways due to activity exerted by the bacterial lipopolysaccharides (LPSs)/endotoxins that further reflect a high risk of miscarriage in various stages. While the presence of some pathogenic entities may be suggestive of an adverse obstetric predisposition, there are still pros and cons of the role of specific strains, as only the vagina and cervix have been targeted as opposed to the endometrium, which recently started to be viewed as the key player in the vagina–cervix–endometrium route. Consequently, based on an individual’s profile, diet, and regime, antibiotics and probiotics might be practical or not. Conclusions: Resident bacteria have a dual facet and are beneficial for women’s health, but, at the same time, relaying on the abundance, richness, and evenness that are definitory indexes standing as intermediaries of a miscarriage.

## 1. Introduction

The intimate and highly specialized relationship between colonizing pathogenic, commensal, and symbiotic microorganisms known to exert a profound impact upon homeostasis since birth through complex networks is a concept that dates back before the culmination of the Human Microbiome and Integrative Projects ((i)HMP) [1,2,3]. This topic paved the way and ensured a transition to a novel dimension by offering advanced research and clinical perspectives into the microbiome’s position in the overall development, health, interplay with the immune system, and disease [4,5,6].

The advancement and constant accessibility of high-throughput sequencing techniques facilitate our knowledge, thus becoming tangent with outstanding research and filling distinct niches [4,7,8,9]. In this context, molecular biology contributed by bringing forward the concept of “microbiocenosis” and identification of anaerobic Gram-negative and Gram-positive entities [10] regarded as etiological factors in obstetric and gynecological complications [11,12].

Even though the translation of intricate ecological metrics into clinical trials remains problematic and poses a great challenge, a classifying system would be of great importance, leading to an accurate data set [13,14], as in other studies, to create community structure type (CST) clusters [15]. Based on the system regarding the five CSTs proposed by Ravel and colleagues [16], four are dominated by *Lactobacillus* spp. as follows: CST I by *Lactobacillus crispatus*, CST II by *Lactobacillus gasseri*, CST III by *Lactobacillus iners*, and CST IV by *Lactobacillus jensenii* that is otherwise loomed with over 25% by anaerobic bacteria [17]. Conversely, others proposed that machine learning algorithms ensure a prospect in order to overcome classifying discrepancies [18] and discussed how bioinformatics analyses ease the working volume to make the microbial community groups independent and comparable [19,20].

To be precise, the role of maternal microbiota started to gain attention from researchers [21,22,23] owing to the nonsterile status of the womb and bacterial colonization of the uterine cavity, presumably derived following the ascension route of bacteria from the vagina through the cervical canal [24,25,26]. Nevertheless, current working protocols and sampling methods (double-sheathed embryo transfer—ET, catheter, or transcervical device) aiming to minimize contamination provided a comprehensive overview of the communities within the endometrium [27,28,29] in contrast with culture- and microscopy-based procedures [30,31,32].

As a first-line protection against foreign entities and responsible for a dynamic balance between interdependence and mutual restriction, the vagina is a unique habitat for a broad spectrum of microorganisms and is vital [33,34,35] for conception and pregnancy, as well as a passage for sperm, menstrual blood, and childbirth [36,37,38,39,40]. However, over the course of a lifespan, the composition may vary based on a series of exogenous factors such as age, ethnicity, and geographical location [41].

Vaginal flora composition is similar to the endometrium, being dominated by *Lactobacillus* spp. involved in lactate, bacteriocins, and hydrogen peroxide production, and by other genera [16,28,29,42]. This link is partly assured by elevated estrogen levels that facilitate glycogen deposition in the vaginal epithelium cells [16,43,44,45,46] and by microbiota that intertwines with the existing microenvironment [47].

In contrast, whereas the overall diversity and richness decrease throughout gestation and stability increases due to the elevated abundance of *Lactobacillus* to prevent pathogen adherence and reducing pH in contrast with nonpregnant women, there are numerous endo- (menarche, menses, menopause, sexual activity, pregnancies, antimicrobial agents) and exogenous (stress, diet, exercise, hygienic routine, use of contraceptives, and rectal colonization) factors shaping composition [40].

Therefore, a possible alteration and depletion of lactobacilli no longer maintain the optimal conditions for carrying a baby and the host’s eubiosis and might be a pillar of dysbiosis and a forerunner of poor pregnancy outcomes [48]. A number of studies have been published throughout the years that greatly expanded the sphere of information with emphasis on infections (herpes simplex virus 2 (HSV-2), human immunodeficiency virus (HIV), human papillomavirus (HPV), aerobic vaginitis (AV), and bacterial vaginosis (BV)) [49], as well as more complex conditions that include gestational diabetes, intrauterine adhesions (IUAs), polycystic ovary syndrome (PCOS), recurrent implantation failure, preterm birth (PTB), preterm pre-labor rupture of fetal membranes (PPROMs), early miscarriage, infertility, and cancer [25,50,51].

Despite the recent advances, some etiologies and pathogenesis that might occur are yet to be explored to their complete potential in relation to microbiota. Cumulatively, the present manuscript follows to discuss in an objective and possible critical manner all existing studies with regard to the lower female genital tract (FGT), particularly the vagina and cervix, and the endometrium within the upper FGT with the most challenging issues in reproductive medicine.

## 2. Methodology

The structure and design of this manuscript adhere to the standard procedures, instructions, and indications established previously by Green et al. [52] on writing a narrative review.

### 2.1. Academic Databases Accessed and Search Strategy

We conducted a comprehensive search of the scientific literature aiming to identify, gather, and sort available studies within the field, utilizing four well-known academic databases: PubMed, ISI Web of Knowledge, Scopus, and ScienceDirect. Using a controlled vocabulary and scientific language, we ensured the discovery of relevant articles that align with our objective.

The analyseswere carried out using “microbiome” as a primary dimension alongside “vagina”, “cervix”, and “endometrium”; as a second output, “early/late miscarriage”, “early pregnancy loss”, “spontaneous abortion”, “missed abortion”, “recurrent miscarriage”, and “recurrent pregnancy loss” were used.

We have concentrated solely on studies conducted on human individuals.

The adopted PubMed string was: microbiome [Title/Abstract] AND vagina [Title/Abstract] AND cervix [Title/Abstract] AND endometrium [Title/Abstract] AND early miscarriage [Title/Abstract] AND late miscarriage [Title/Abstract] AND early pregnancy loss [Title/Abstract] AND spontaneous abortion [Title/Abstract] AND missed abortion [Title/Abstract] AND recurrent miscarriage [Title/Abstract] AND recurrent pregnancy loss [Title/Abstract].

The adopted ISI Web of Knowledge, Scopus, and ScienceDirect strings were: microbiome [Title/Abstract] AND vagina [Title/Abstract] AND cervix [Title/Abstract] AND endometrium [Title/Abstract] AND early AND miscarriage [Title/Abstract] AND late AND miscarriage [Title/Abstract] AND early AND pregnancy AND loss [Title/Abstract] AND spontaneous AND abortion [Title/Abstract] AND missed AND abortion [Title/Abstract] AND recurrent AND miscarriage [Title/Abstract] AND recurrent AND pregnancy AND loss [Title/Abstract].

### 2.2. Inclusion and Exclusion Criteria

To complete the initial evaluation step, studies had to meet specific criteria. They had to be original experiments written in English and published between 2010 and May 2023. Only those that exclusively enrolled female patients were considered. Any literature synthesis, unavailable content, inclusion of male patients, or studies conducted on experimental models have been eliminated.

### 2.3. Study Selection

The papers that potentially met the criteria were assessed by examining their titles and abstracts. Subsequently, the entries were thoroughly evaluated based on the content, and any discrepancies or conflicting viewpoints were resolved through the joint approval of two expert specialists, B.D. and C.I.

### 2.4. Number of Entries

**Figure 1:**
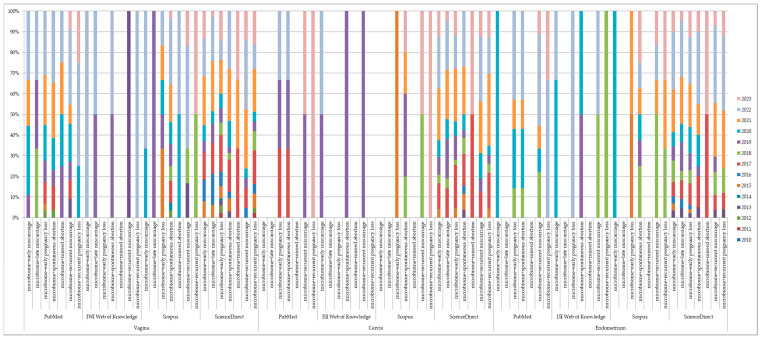
Cluster presenting the studies published from 2010 until 2023 on PubMed, ISI Web of Knowledge, Scopus, and ScienceDirect, based on the combination of keywords and in relation to each segment of interest along the female reproductive tract.

Over the past thirteen years, we found a total of n = 804 results for all main keywords across different databases. The breakdown per database is as follows: n = 122 (15.17%) in PubMed (n = 86, n = 9, and n = 27), n = 22 (2.73%) in ISI Web of Knowledge (n = 8, n = 5, and n = 9), n = 77 (9.57%) in Scopus (n = 48, n = 9, and n = 20), and n = 583 (72.51%) in ScienceDirect (n = 255, n = 155, and n = 173), as shown in Figure 1.

**Figure 2:**
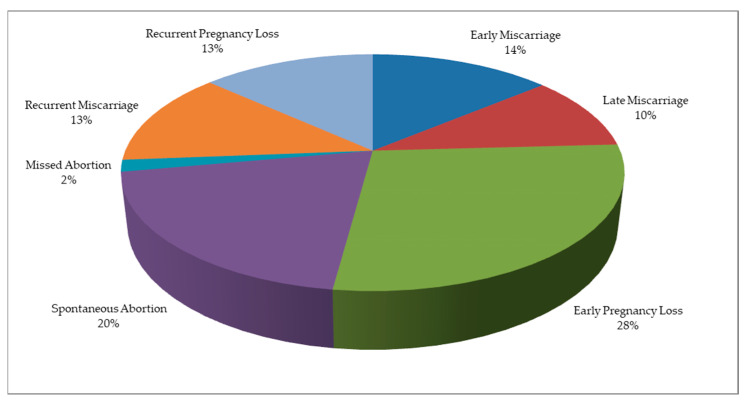
Percentages of studies allocated to targeted adverse obstetric outcomes.

Depending on the trimester when the pregnancy ceases from evolution, we identified: n = 112 studies for “early miscarriage”, n = 82 for “late miscarriage”, n = 225 for “early pregnancy loss”, n = 161 for “spontaneous abortion”, n = 13 for “missed abortion”, n = 104 for “recurrent miscarriage”, and n = 107 for “recurrent pregnancy loss”, as presented in Figure 2.

**Figure 3:**
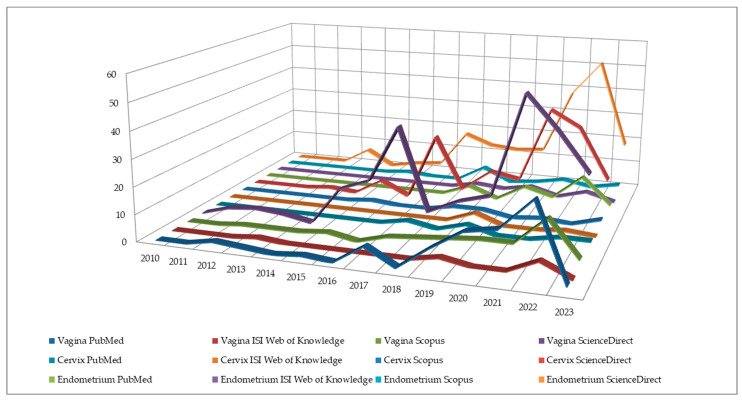
Trendline of the studies published between 2010 and 2023 based on the female reproductive tract per each database.

In a retrospective manner per year of published study, n = 0 in 2010, n = 3 in 2011, n = 7 in 2012, n = 13 in 2013, n = 2 in 2014, n = 28 in 2015, n = 23 in 2016, n = 95 in 2017, n = 48 in 2018, n = 68 in 2019, n = 76 in 2020, n = 165 in 2021, n = 198 in 2022, and n = 78 in 2023 until May, as Figure 3 suggests.

### 2.5. Number of Results

After eliminating the duplicates, n = 84 manuscripts met the eligibility criteria and were considered for further evaluation following the first phase fulfillment. However, we pulled n = 16, from which n = 6 were reviews, n = 2 were study protocols, n = 2 included animal models (n = 1 on cows and n = 1 on mice), n = 2 were out of the scope, n = 1 could not be adequately evaluated, n = 1 was a case report, n = 1 was an article written in Chinese, and n = 1 was an editorial. After the second stage, only n = 22 out of the n = 68 studies were considered appropriate for inclusion in the main body of this manuscript.

An overview of all studies can be observed in Table 1.

## 3. Results and Discussion

It has been observed that microbial ratios of the endometrium can fluctuate in both healthy individuals [27] and those experiencing menorrhagia [77]. This characterizes an increase in *Lactobacillus* during distinct phases of the menstrual cycle and a low α-diversity [78]. Vomstein et al. [58] examined the longitudinal intra-cycle changes in the follicular, ovulatory, and luteal phases, and found a decrease in the richness and evenness of species in healthy women during ovulation, with the lowest load in the luteal phase after the calculation of the Shannon index. However, this finding did not apply to RM or RIF groups, instead noting a higher similarity marked by an increase in *Firmicutes* and a decrease in *Proteobacteria* in the reference group compared to RIF and *Firmicutes* in the RM [58]. Even though this study did not emphasize significant changes in the uterine microbiota at days 2 and 7 of the menstrual cycle attributable to the ovulatory and luteal phase, Vomstein et al. [58] still noted subtle translocations that support the hypothesis of a stabilizing flora related to the evolution of the menstrual cycle. With a decrease only in the control group, the endometrium seems to provide proper conditions for proliferation, and its thickness might be used as a predictor of reproductive success [79,80].

It is possible that an imbalance in RM and RIF patients who have low levels of *Lactobacillus* spp. and have experienced a pregnancy [81] or undergone a controlled ovarian stimulation (COS) protocol could be a contributive factor for endometrial and vaginal dissimilarity in favor of *Prevotella* and *Atopobium* [82]. Although RIF and RM are common in nulliparous women, research concentrated on endometrial microbiota is limited [54,83] and has produced mixed results, completed by others that failed to demonstrate the correlation with reproductive success [64,83,84,85]. *Proteobacteria* and *Dialister* may have a direct impact on reproductive function in women with RIF, further associating with endometriosis (EMS) and chronic EMS [86]. This may represent a potential marker for dysbiosis in this category of patients [87]. Antibiotics are currently the only option to improve the live birth rate (LBR) in chronic EMS as a countermeasure to probiotics’ low efficiency [88,89,90,91]. Multiple routes for microorganism ascension have been proposed over the years. The two options point to endometrial colonization that portrays hematogenous colonization of the oral and placental flora [92], whereas the most accepted remains that through the cervix. Uterine microbiota via the cervix regulates the traffic between the sites, and the mucus plug serve as an indicator of infection [93], shaped by the lactic acid bacteria strains’ pH transitions [27,94]. Gonadal hormones influence the composition and function of the vaginal microbiota [95] with *Lactobacillus* spp. being prevalent in women with chronic EMS or endometrial polyps [96]. Additional data ascertain the presence of a positive and uneven distribution of the endometrial microbiota in RM and endometrial polyp chronic EMS patients [96,97].

The use of combined oral contraceptives (COCs) and levonorgestrel intrauterine system (LNG-IUS) can support the growth of *Lactobacillus* spp. during the follicular and luteal grades, as it correlates with the menses [98]. Some studies investigate the effectiveness of balloon placement versus intrauterine device (IUD) for patients who aborted or had intrauterine adhesions (IUAs), as there is no significant difference in ongoing pregnancy rates between the two groups. However, the patients who underwent balloon placement had a lower miscarriage risk, but with no differences in terms of IUA recurrence, American Fertility Society (AFS) score reduction, or chronic endometritis rates, and an increase in bacterial load compared to those who used the balloon stent [99]. Though the mycobiome remains underexplored, there is no significant difference between the groups, except for an increase in *Dialister*, *Filobasidium*, and *Exophiala*, and a decrease in *Bifidobacterium* in IUA patients [100].

From current knowledge, it is clear that *Lactobacillus* spp. fulfill a vital role in safeguarding the vagina in the early stages of pregnancy. Among them, *Lactobacillus crispatus* is particularly important. To understand the importance of *Lactobacillus* species, it is essential to note that glycogen is broken down into small polymers and then metabolized to produce D-lactic acid, bacteriocins, and hydrogen peroxide. These synthesized compounds confer protection against the growth, establishment, and proliferation of pathogens that cause infections [95,101,102]. *Cutibacterium* and *Anaeroba* have been identified as predictors of miscarriage in patients with recurrent pregnancy loss (RPL). Despite *Cutibacterium*’s dominance in the skin, it has also been found in the uterine endometrium [103] and in follicular fluid [104], where it can exert detrimental effects when present with *Bifidobacterium*, *Gardnerella vaginalis*, *Atopobium*, and *Lactobacillus* (though not *Prevotella*) based on the geographic location [105]. While oral probiotics were found to be ineffective in altering the vaginal microbiota [106], a reevaluation of the cervicovaginal microbiome showed an increase in the interleukin 6 (IL-6) in patients predisposed to RPL [107]. This discovery could prove to be useful in determining which patients are suitable for progesterone (P4) therapy [108].

When certain bacteria are depleted, it cause an increase in *Streptococcus*, *Peptoniphilus*, *Ureaplasma*, *Dialister*, *Prevotella*, *Bacteroidetes*, and *Firmicutes* [109]. These stand as triggers of inflammation, which in turn enhances the up-regulation of metalloproteinases [110,111] and pro-inflammatory cytokines [112], disturbing iron processing and decreasing the red blood cells (RBCs). In severe cases of inflammation or bleeding [113,114], tissue inhibitors of metalloproteinases (TIMPs) [115] are inhibited, thus exacerbating the severity. Miscarriage can be linked to hormonal signaling dysregulation between estradiol (E2) and P4 levels with substrate availability [116,117], which causes a drop in *Lactobacillus* spp. levels. Estrogen promotes glycogen accumulation in the vaginal cells, for which *Lactobacillus* spp. are exploiting it as a carbon source [16]. The ratio of *Firmicutes* and *Bacteroidetes* increases in women who miscarry [118], indicating a holistic dysbiosis of the rectal microbiome. This relationship is primarily influenced by host factors such as age and gestational weeks, rather than the action of a specific pathogen [119].

Women with *Lactobacillus* spp.-dominant flora have higher rates of successful implantation, pregnancies, and LBR after in vitro fertilization (IVF)-ET compared with those with non-*Lactobacillus*-dominant flora, such as [29] as *Klebsiella*, *Neisseria*, *Bifidobacterium*, and *Gardnerella* [120]. Interestingly, the microbiota of the uterine endometrium does not seem to have any impact on the IVF-ET outcomes of infertile women [85] or pregnancy/miscarriage rates [84,121]. To ensure the utmost accuracy of sample collection, it is recommended to use an ET catheter [53,54] during clinical practice to prevent contamination with harmful bacteria from the cervical canal that may affect the embryo [122,123].

Although *Lactobacillus crispatus* is regarded as a part of a healthy microenvironment [124], it is less abundant in women with EMS [87] and RPL [67] in comparison to *Lactobacillus iners* in the endometrium of RPL women and *Gardnerella vaginalis*. Peuranpää et al. [67] highlighted a varying ratio of *Lactobacillus crispatus* and *Gardnerella vaginalis* in RPL and healthy women.Despite the limited evidence demonstrating a relationship between the microbiome and RPL [55,65,67], a correlation between the PTBs and the cervicovaginal microbiota is known [125,126]. *Lactobacillus crispatus* in the vagina correlates with autophagy from the epithelial cells, and low levels of mediators, epithelial p62 with *Gardnerella*, and stress-related 70 kilodalton heat shock proteins (hsp70) lead to *Lactobacillus iners*, *Streptococcus* and *Bifidobacterium* dominance [127,128]. Standard laboratory protocols targeting vitamin D deficiency, mid-luteal P4, and chronic endometritis [129], molecular techniques directed in sequencing the 16S rRNA gene, and IVF/IVF-intracytoplasmic sperm injection (ICSI) can help predict microbiome composition alterations. In this context, a forecast of a clinical pregnancy can be made [130], as well as with RIF patients, as the endometrium is colonized by *Streptococcus*, *Staphylococcus*, *Neisseria*, *Klebsiella*, *Phyllobacterium*, and *Sphingomonas* [131,132,133]. The latter two are involved in carbohydrate and fat metabolism by regulating the immune cells’ Th17 and ratio of Th1 and Th17, based on the synthesized LPSs [118,133]. This further explains the chronic endometritis [134] due to the pro-inflammatory landscape and the relative abundance of *Prevotella_1*, *Prevotellaceae_UCG_003*, and *Selenomonas_1*, as well as the correlations of imidazolepropionic and 1,4-methylimidazoleacetic acids with recurrent miscarriages [118].

A study by Garcia-Grau et al. [53] reported the case of a woman who underwent several ET cycles that resulted in RRF. Investigations and medication that included antibiotics and probiotic vaginal tampons indicate virulence genes implicated in biofilm and antibiotic resistance of *Gardnerella vaginalis*, documented to cause dysbiosis [135]. Complementary to these observations stand much more severe complications, such as subfertility [136], in early [29,53,137], late [138], and spontaneous miscarriage [81]. It is noteworthy that genital flora is dynamic and can be manipulated by external factors, such as the body mass index (BMI) adjusted to the RPL group [67] and further translocations that occur with aging. It is relatively stable in the 20–40 interval, and alterations arise after 50 years [139]. Despite the best efforts, the mechanisms behind a more diverse microbiota are still not fully deciphered [140,141,142] and connected to cytokine level disturbances [57]. In vitro experiments showed that *Lactobacillus crispatus* prevents the attachment of pathogens by binding to the decidualized endometrial cells [143].

Endometrial dysbacteriosis weakens the tight junctions, which leads to the adherence of pathogens to endometrial stroma [140]. The Toll-like receptors (TLRs) current on the surface of endometrial cells are activated, resulting in localized immune reactions [142]. This mixed effect can cause remodeling of spiral arteries [144] and disruptions of the placenta due to the inadequate development of the natural killer (NK) cells that are vital in trophoblast invasion [145]. Subsidiary studies have shown a unique flora reuniting thirteen taxa in the endometrial tissue, eight in the fluid, and twenty-two different in abundance. The four genera frequently encountered are *Flavobacterium*, *Achromobacter*, *Exiguobacterium*, *Brevundimonas*, and phyla *Verrucomicrobiaceae*, according to Liu et al. [54]. Multivariable logistic regression analyses have emphasized that *Ureaplasma* spp. and prior miscarriages are independent risk factors for euploid karyotype, but related to preterm delivery in prospective gestations, according to Shi et al. [65].

Through the 16S rRNA sequencing of specific hypervariable regions, over four hundred patients were categorized into groups, leading to significant discoveries [18,59,68,70]. *Lactobacillus* spp. was the predominant species in both the cervix and vagina, but its prevalence decreased in cases of first-trimester miscarriage [18], and RM [59], according to Al-Memar, Caliskan, and their collaborators. The gestational age and RBCs count are responsible for the fluctuations in the α-diversity, as indicated by Guang et al. [68], as it extrapolates in richness and hierarchical clustering analysis [18,68]. Mori et al. [70] strengthen the abovementioned pointing toward the prevalence of *Lactobacillus* spp., especially *Lactobacillus iners*, and several pathogens. *Gardnerella vaginalis*, *Atopobium vaginae*, and *Bifidobacterium breve* arebelieved pivotal for the host’s eubiosis. Patients with either RPL or a history of chorioamnionitis or miscarriage exhibit a separate microbial pattern compared to controls. Specifically, in terms of *Delftia* in the RPL and chorioamnionitis and *Streptococcus*, *Chloroplast*, *Microbacterium*, *Delftia*, *Anaerobacillus*, and *Cutibacterium* in the chorioamnionitis, with miscarriage groups containing the last two genera [70].

Recent research has firmly established a causality link between a prior growth of certain pathogens, especially *Atopobium*, and first-trimester spontaneous abortion (SA). This correlation is reflected in the β-diversity of the analyzed sites. *Proteobacteria* and *Pseudomonas* may be responsible for the cases of RSA compared to women who suffered a natural pregnancy-induced abortion, as suggested by Seo, Liu, Fan, et al. [57,63,73]. Demographic data [73] and published papers indicate that the highest percentage of cases occurs in women aged 45–54 years old, with poor education, an unmarried status, and a history of pelvic inflammatory disease (PID) [146]. There is a current debate about the possible connection between SA and human papillomavirus (HPV) [147]. High-risk (HR)-HPV oncogenic basis has previously been addressed [73]. The proposed passages for this relation include alternative proof that HPV is accountable for the apoptosis process of infected trophoblasts, which negatively impacts implantation and placental physiology [148,149,150,151].

A regulated intake of *Ligilactobacillus salivarius* CECT5713 significantly enhances the reproductive prospects, according to Fernández et al. [62]. Otherwise, it can culminate in an increase in *Atopobium vaginae*, *Sneathia sanguinegens*, and *Leptotrichia amnionii* [73]. Analyses carried out targeting *Ligilactobacillus salivarius* CECT5713 underlined its antimicrobial action against *Gardnerella vaginalis*, *Streptococcus agalactiae*, *Candida albicans*, *Candida glabrata*, *Candida parapsilosis*, and *Ureaplasma urealyticum*. This capability is facilitated by its ability to build L-lactic acid and acetic acid molecules [152] responsible for the transition of the host’s dysbiosis-to-eubiosis state [153,154,155]. One key benefit of *Ligilactobacillus salivarius* CECT5713 is the activity of inactivating pathogens found at the reproductive tract level [156,157,158,159,160,161,162]. Its viability depends on the low pH to hamper the growth in the local vaginal biofilm [163,164]. Another strong point is the vaginal cells’ adhesion rate and co-aggregation with vaginal pathogen *Streptococcus agalactiae* [156] or *Candida* spp., interconnected to the binding sites [165,166] that are strain-specific characteristics [156,167,168,169]. Approximately 9 log_10_ CFU/day for half a year increases the chances of a successful pregnancy up to 56% [62]. However, there is some controversy surrounding former observations that suggest a persistent bacterial richness of *Lactobacillus* among the groups enrolled [57] and later contradicted in another manuscript [73].

Irrespective of the *Cutibacterium* and *Staphylococcus* spp. as part of the core microbiota [57,103], under typical homeostasis circumstances, alteration facilitates an interchangeable phenomenon of bacterial load complementary to the causative facet in repetitive abortions. This shift in microbial abundance triggers an in-chain reaction that disturbs the cytokine landscape. It activates the IL-6 and gamma interferon (IFNγ) [57] and further confirms the hypothesis of an increase in chemokine (C-C motif) ligand (CCL) 2/3/4/5/8 cytokines in villus tissues [63] knowing that *Bacillus pumilus* could direct the messenger RNA (mRNA) up-regulation of IL-1α, IL-6, IL-8, and of C-X-C motif chemokine ligand 1-3 (CXCL1-3) in inflammation [170,171]. Women who have experienced RSA reunite *Corynebacterium*_1, *Burkholderia*-*Caballeronia*-*Paraburkholderia*, *Sphingomonas*, *Rhodococcus*, *Megasphaera*, *Sneathia sanguinegens*, and *Pseudomonas*, as in [63], and can be re-drafted following aspirin and metformin administration to restore *Lactobacillus* spp. balance, based on a study from Zhao et al. [72]. Recent publications suggest personalization among the constitutive niches. Therefore, studies accentuate the tendency of microorganisms to migrate and illustrate the acidic pH and Nugent score. These are completed by the abnormalities of the immune transforming growth factor beta 1 and 2 (TGF-β 1/2), and vascular endothelial growth factor (VEGF) levels [62]. This notwithstanding, a low pH and Nugent score correlate with the high concentration and dominance of lactobacilli [16,172,173], which occasions that exemplified the consequences of uncontrolled antibiotics administration across various stages of life. It is counterproductive for the lactobacilli population [174] and provoke fertility impairment or embryo implantation failure [62]. *Lactobacillus crispatus*/*gasseri*/*iners*/*jensenii* are predominant in the vagina [62], with the mention that is less common or absent in other habitats [16,175,176]. Assuming that a co-dominance of multiple species in the community is not typical [176], *Lactobacillus crispatus* has been positively associated with the reproductive outcomes after treatment compared to *Lactobacillus iners*/*gasseri*, unless *Lactobacillus salivarius* ascended as the dominant species. Fluctuations between these strains are not uncommon [177], and their respective roles have been extensively studied [176,177,178,179,180]. On the other hand, others maintain the role of *Lactobacillus crispatus* on reproductive health [181,182,183], contrary to *Lactobacillus iners* that presumably accomplish dual functions for vaginal health [184,185] or to induce translocations [124,135,178,180,186].

As we explore this topic further, conflicting reports [55,56] arise regarding the microbial profile similarities of the α-diversity and β-diversity. There has been noted a decline in *Bifidobacterium* spp. to the detriment of an increase in *Gardnerella* spp. in the reference group and *Atopobium* spp., as formerly shown in the RSA group [73], as these microbes are documented in advancing uterine and peripheral NK cell counts [55,63,76]. Sialidases are enzymes documented to catalyze the subtraction of sialic acid from eclectic glycoconjugates found in both *Prokaryota* and *Eukaryota* domains that are essential for the pathogenesis and nutritional requirements of pathogens. Vaginal sialidase activity is persistent in all samples as in the cases of BV [187], thus implying the leukocyte esterase (LE) inflammatory indicator for urogenital tract and periprosthetic joint infections [188,189], which was statistically higher in the RSA group [72]. Kuon et al. [76] suggest combining conventional and molecular protocols without 16S rRNA sequencing and show a prevalence of vaginal infections-related entities in the RM group. They found a high colonization of *gram-negative anaerobes* (20.5%), *Gardnerella vaginalis* (19.0%) through the elevated peripheral natural killer (pNK) cells, *Enterobacteriaceae* (14.8%), group B *Streptococcus* (11.0%), and *Candida* species (7.9%), coupled with the absence of *Lactobacillus* spp. (14.5%), but *Chlamydia trachomatis* was detectable in only 0.53% [76].

Recent data has unequivocally demonstrated that *Lactobacillus* sp. partakes in maintaining women’s health by the amount of lactic acid and hydrogen peroxide to suppress the proliferation of pathogens such as *Mobiluncus* sp. *Bacteroides*, *Prevotella*, and *Gardnerella vaginalis* [190,191], which is responsible for BV. Conversely, a sub-optimal abundance of *Lactobacillus inners*/*crispatus* and *johnsonni* ratios and an interchange with *Atopobium vaginae*, *Aerococcus christensenii*, *Leptotrichia amnionii*, *Prevotella amnii*, *Lactobacillus fornicalis*, *Ureaplasma parvum*, *Mycoplasma hominis*, and *Sneathia sanguinegens* can result in severe pregnancy-related complications. These vary from BV to preterm delivery, abortion, and chorioamnionitis [192,193,194], and even a decline in the secretion of leukocyte protease inhibitor (SLPI) in the vagina [195]. Metronidazole is a first-line drug prescribed for treating BV, but includes its limitations. While it is effective in the short term, a relapse occurs in 20% of patients with BV within one month [196] and up to 58% within one year [197]. Additionally, resistance to the drug can develop, with a range detection of 75–100% in women with recurrent BV after a therapeutic administration [198], which can be attributed to variable susceptibility or intrinsic feature [199]. Probiotics [200] and synthetic compounds like amoxicillin/clavulanic acid or clindamycin have been reviewed as alternatives, but there are concerns regarding dose and duration [201].

In standard clinical settings, when anaerobic microorganisms replace *Lactobacillus* spp. [202], diagnosis methods involve Nugent’s scoring system [203] or Amsel’s criteria [204]. To manage the risk of second-trimester loss and PTB [138,205,206], international guidelines recommended screening programs [207,208,209], even for women with a low risk of miscarriage due to the absence of *Lactobacillus* spp. [210]. Certain socio-demographic characteristics, age, clinical history, and *Gardnerella vaginalis* among populations can cause abnormal vaginal flora in women with RPL, BV, or recurrence of BV, increasing the risk of experiencing adverse pregnancy outcomes from 20 weeks to less than 37 weeks [211,212,213]. Prior to BV manifestations, a conversion occurs, leading to a dominance of *Lactobacillus iners* during remission and transitory episodes, but ultimately benefiting other microorganisms in the pre-conversion phase [214]. Although personalized treatment guidance based on probiotics and antibiotics may reflect the IVF and reproductive developments in succeeding ET cycles [131,132], it cannot prevent BV in pregnant women [106].

*Bifidobacterium*, similar to certain *Lactobacillus* spp. has a marked impact on the microenvironment [57]. Studies of microecologies in the vagina and cervix have led to the conclusion of dominant *Firmicutes* and *Actinobacteria*, with slight differences in the vaginal *Lactobacillus*, and minor ones in the relative abundance of *Bifidobacterium* and *Gardnerella* spp. in the vagina. In a group that underwent RSAs, high levels of several bacteria were found, including *Bacteroidetes*, *Crenarchaeota*, *Cutibacterium*, *Atopobium*, and *Staphylococcus* spp., with a tendency of increase in the last three genera [57]. A recent publication demonstrated a relative abundance of *Bacteroides*, *Tenericutes*, *Fusobacteria*, *Proteobacteria*, and *Firmicutes*, analogous to those from cervical intraepithelial neoplasia (CIN) samples [215] and higher operational taxonomic units (OTUs) in the non-abortion group in comparison to induced abortions or SAs [73]. Possible contributing factors for cervical microbiota dysbiosis in the non-abortion group as compared to SA include individual hygiene practices, genetic factors, glycogen level, or the method used for sample collection and sequencing [16,216,217].

Research to explore the association between a disrupted microbial flora and missed abortion reveals on the computational bioinformatic cladograms an altered α-diversity based on the Simpson index and high Shannon index in the patient’s group, according to Sun, Liu, Oliveira et al. [61,69,71]. The relative abundance of *Firmicutes*, *Lactobacillus crispatus*, *jensenii*, and *gasseri* and, intriguingly, in *Mycoplasma genitalium* and *Ureaplasma parvum* appeared to be reduced but is counterbalanced by high richness and diversity of *Bacteroides*, *Miscellaneous*-*Crenarchaeota*, *Escherichia*/*Shigella*, *Acetobacter*, *Staphylococcus*, *Bacillus*, and *Halomonas*. They concluded that these modifications were associated with the metabolic function in the case group, having different CSTs and ranging from a dozen to hundreds or thousands of OTUs with a space under the receiver-operatic curve (ROC) of 86.76% and 93.33%. Subsidiary observations referring to this gynecological condition attribute multiple arguments toward a disbalance between *Lactobacillus* and *Gardnerella* as frequently observed events. Moreover, the high prevalence of euploid miscarriages than aneuploid due to dissimilarities in genera *Fam_Finegoldia*, *Lac_Coprococcus_3*, and *Lac_Roseburia* is linked to 80% of embryonic miscarriages, according to Jiao, Grewal, Xu and their collaborators [56,61,75]. These translocations follow a linear reactivity in the up-regulation of pro-inflammatory cytokine levels IL-1β/2/6/8/10/12p70 and an influx of polymorphonuclear cells in placental tissue [56,61,75].

As previously mentioned, a lack of *Lactobacillus* spp. (*Lactobacillus jensenii*, *Lactobacillus gasseri*, but not *Lactobacillus iners*), *Firmicutes* and *Saccharibacteria* can contribute to first-trimester miscarriages. This is due to an increase in the α-diversity of certain genera including *Fam_Finegoldia*, *Lac_Coprococcus_3*, and *Lac_Roseburia*, *Proteobacteria*, *Actinobacteria*, *Chlamydiae*, and *Fusobacteria* [18,60,69], which is also noted in ectopic pregnancies [218]. It is worth noting that women who experience embryonic miscarriage exhibit low levels of IL-10 expression compared to IL-2 in the control group. Vaginal metabolites such as xanthine, benzoate, ascorbate, fumarate, and inosine are predictors. Furthermore, Th2 and regulatory T cells (Treg) are correlated to these secreted cytokines [60,219,220]. Interestingly, there was no *Mycoplasma genitalium*, *Mycoplasma hominis*, and *Ureaplasma* spp. in the patient group [69], but further investigations are necessary to confirm this.

There has been some uncertainty surrounding the role of vaginal flora in determining obstetric outcomes due to the presence of *Ureaplasma* spp., which was found to be similar in both groups analyzed. However, when the lower genital tract is infected with *Mycoplasma hominis*, *Ureaplasma urealyticum*, and *Ureaplasma parvum*, it can lead to placental tissue infection and alter the tolerogenic state, consequently triggering extrinsic apoptotic pathways [71]. It is believed that vaginal dysbiosis is linked to low levels of *Lactobacillus* spp., which can lead to chromosomally normal miscarriages [61,75,221], with premature delivery [222], infertility [223], IVF failure [224], premature rupture of membranes (PROM) [225], PID [226], and RM [56]. This dysbiosis might be correlated with *Fusobacterium*, *Finegoldia*, *Coprococcus*, *Roseburia*, *Atopobium*, and *Prevotella* [18,56,60,61]. Despite some predicting the involvement of vaginal microbiota in adverse pregnancy outcomes from PTB [227,228,229,230] to postpartum complications [231], others argue that the *Lactobacillus* vagitype may not be a factor [232,233].

*Lactobacillus* spp. are key strains that can help the host to prevent cerclage failure [234] and premature cervical dilatation, while *Gardnerella vaginalis* abundance is also reported to be responsible for unsuccessfully rescuing cerclage cases [235], considering that those who had an insertion of a transabdominal cerclage for large loop excision of the transformation zone (LLETZ) or a cone biopsy results in a high rate of live births [236]. Depletion of *Lactobacillus* spp. and the shifts that occur may be due to post-pregnancy IVF-ICSI treatment [130], infertility [64] of known or unknown origin, and RPL [237]. The analyses of samples collected from women who underwent a total hysterectomy with bilateral salpingo-oopherectomy present a high abundance of *Firmicutes* and *Lactobacillus* [238]. Besides the non-*Lactobacillus*-dominated niche [64], patients display distinctive profiles of the menstrual blood-NK subtype [237]. A recent method illustrates a fast and inexpensive diagnostic tool to determine culturable and non-culturable microorganisms in patients with infertility and EMS [239]. Nonetheless, others reveal no distinctions between *Lactobacillus*-dominant (>80%) and non-dominant (<80% and >20% opportunistic entities; e.g., *Atopobium*, *Gardnerella*, *Streptococcus*, *Staphylococcus*, *Corynebacterium*, and *Bifidobacterium*) endometria on pregnancy and miscarriage rates [84,240]. A recent study presents the case of a woman who experienced a spontaneous miscarriage in the eighth week and subsequently during a successful pregnancy. As discussed above, there was a high bacterial diversity and a low *Lactobacillus* abundance, notably translocations between *Lactobacillus crispatus* and *Lactobacillus iners* in relation to PTB, prior to SA, scheduled, or before birth [81,241,242]. Conversely, another publication opposes these results and supports a sporadic presence of *Lactobacillus iners* and *Lactobacillus crispatus* in the middle endometrium, but rather being colonized by *Acinetobacter*, *Pseudomonas*, *Cloacibacterium*, and *Comamonadaceae* [243].

Several studies discussed race/ethnicity and sample location as probable causes for PTB irrespective of the usual depletion of *Lactobacillus* and the increase in putative novel bacteria. These changes in load proved not to be correlated with PTB [244], subsequent data rejecting this since a positive *Ureaplasma* in the vagina can cause preterm delivery, especially in women with threatened premature labor [245]. This is probably correlated with the vaginal level of β-defensin-2, as it reduces the risk of spontaneous PTB [233]. The bacterial DNA signatures of *Gardnerella vaginalis* (clade 4), *Lactobacillus iners*, and *Ureaplasma parvum* (serovars 3 and 6) through the algorithms (GLU test) developed may identify women with a singleton at high risk of spontaneous PTB at less than 34 to 37 weeks of gestation [246]. Even though PPROM is common among women [246], community disturbances occur before the PPROM in females at low and high risk and in only 3% of those who delivered at term [247]. Therefore, an increase in the abundance of *Prevotella*, *Peptoniphilus*, *Streptococcus*, and *Dialister* could be considered an early sign of PPROM [247], while a reduction in *Bacteroides* that is, however, detectable in the first two weeks in both vaginally-born and Caesarean (C)-section delivered infants, demonstrate the mother-to-fetal transmission [248].

## 4. Conclusions

It can be concluded that the reproductive tract microbiome is a complex and intertwined network whose overall integrity and functionality require a relatively low abundance of species, especially representatives of the four CSTs. Independently of the crucial roles and imperative presence of *Lactobacillus crispatus*, *gasseri*, *iners*, and *jensenii*, and of potentially pathogenic in a state of eubiosis as part of the core microbiota, an ascension route of microorganisms from the vagina through the cervix to the endometrium has been theorized. These lactic acid bacteria strains play a central part in synthesizing agents that prevent pro-inflammatory processes in cases of dysbiosis but also modulate metabolic and endocrine functions. However, there is still debate about the involvement of each *Lactobacillus* spp. strain in the reproductive success and outcomes that need to be further addressed. This is of utmost importance since several pathogens may be regarded as indicators of the risk of possible adverse obstetric events. Conclusively, the vagina and cervix are niches accessible to investigate, while the data on the endometrium is scarce due to the high chance of also collecting other bacteria from the reproductive tract. Apart from a woman’s profile, desiring to naturally remain pregnant and successfully carry a gestation or, in some circumstances, through assisted reproductive technology (ART) interventions, it should be emphasized that each individual possesses their own personalized flora. There are a multitude of endo- and exogenous factors shaping the sites and reflects in the lack of antibiotics and probiotics regimes efficiency. We can decipher the mechanisms responsible and map the common species by delineating and dissolving all contradictions and diverging results offered by various teams over the years. In conclusion, the reproductive tract microbiotas have dual facets as both ally and foe. Thus, additional data are compulsory to advance and begincomprehending its complexity and full potential.

## Figures and Tables

**Table 1:** Brief presentation of the studies considered eligible arranged based on the total number of patients and the subsequent allocation, hypervarible region, primers, and sequencing platform.

Total Number of Patients	Allocation of Patients	Hypervariable Region	Primers	Sequencing Platform	Reference
n = 1 patients	n = 1 RRF	V2–4–8 V3–6, 7–9	NS	Ion S5 XL	[53]
n = 25 patients	n = 25 RM	V4	NS	MiSeq	[54]
n = 36 patients	n = 20 Control n = 16 RM	V3–V4	341F [55] 5′-barcode-CCTACGGGNGGCWGCAG-3′ 805R [55] 5′-barcode-GACTACHVGGGTATCTAATCC-3′	MiSeq	[56]
n = 50 patients	n = 25 Control n = 25 RSA	V3–V4	338F 5′-ACTCCTACGGGAGGCAGCA-3′ 806R 5′-GGACTACHVGGGTWTCTAAT-3′	NovaSeq	[57]
n = 50 patients	n = 10 Control n = 20 RM n = 20 RIF	V3–V4	341F 5′-CCTACGGGNGGCWGCAG-3′ 805R 5′-GACTACHVGGGTATCTAATCC-3′	MiSeq	[58]
n = 50 patients	n = 25 Control n = 25 RM	NS	NS	NS	[59]
n = 50	n = 25 Control n = 25 Embryonic miscarriage	V4	515F GTGCCAGCMGCCGCGGTAA 806R GGACTACHVGGGTWTCTAAT	PGM Ion Torrent HiSeq3000/4000	[60]
n = 50	n = 15 Control n = 13 Empty-sac miscarriage n = 22 Missed miscarriage	V4	520F 5′-AYTGGGYDTAAAGNG-3′ 802R 5′-TACNVGGGTATCTAATCC-3′	MiSeq	[61]
n = 58 patients	n = 14 Control n = 21 RA n = 23 INF	V3–V4	S-D-Bact-0341-b-S-17 CCTACGGGNGGCWGCAG S-D-Bact-1290785-a-A-21 GACTACHVGGGTATCTAATCC	MiSeq	[62]
n = 58 patients	n = 27 Normal Induced Abortion n = 31 Unexplained RSA	V3–V4	338F 5′-ACTCCTACGGGAGGCAGCA-3′ 806R 5′-GGACTACHVGGGTWTCTAAT-3′	MiSeq	[63]
n = 63 patients	n = 44 Became pregnant n = 19 Did not become pregnant	V4	515F [64] 5′-TCGTCGGCAGCGTCAGATGTGTATAAGAGACAGGTGYCAGCMGCCGCGGTAA-3′ 806rB [64] 5′-GTCTCGTGGGCTCGGAGATGTGTATAAGAGACAGGGACTACNVGGGTWTCTAAT-3′	MiSeq	[65]
n = 85 patients	n = 39 Control n = 46 RPL	V3–V4	341F [66] 5′-CCTACGGGNGGCWGCAG-3′ ITS1F [66] 5′-GGTCATTTAGAGGAAGTAA-3′ 785R [66] 5′-GACTACHVGGGTATCTAATCC-3′ ITS2 [66] 5′-GCTGCGTTCTTCATCGATGC-3′	MiSeq	[67]
n = 87 patients	n = 24 Elective Abortion n = 63 Missed Miscarriage	V4	515F 5′-GTGCCAGCMGCCGCGGTAA-3′ 806R 5′-GGACTACHVGGGTWTCTAAT-3′	Mini Seq	[68]
n = 104 patients	n = 50 Control n = 54 Missed abortion	V1–V9	F44 RGTTYGATYMTGGCTCAG R1543 GGNTACCTTKTTACGACTT	Bacterial chip	[69]
n = 105 patients	n = 17 Control n = 88 Unexplained RPL	V3–V4	S-D-Bact-0341-b-S-17 5′-CCTACGGGNGGCWGCAG-3′ S-D-Bact-0785-a-A-21 5′-GACTACHVGGGTATCTAATCC-3′	MiSeq	[70]
n = 109 patients	n = 20 Control n = 89 SA	NS	NS	NS	[71]
n = 126 patients	n = 18 Control n = 108 RSA	V3–V4	338F 5′-ACTCCTACGGGAGGCAGCAG-3′ 806R 5′-GGACTACHVGGGTWTCTAAT-3′	MiSeq	[72]
n = 147 patients	n = 23 SA n = 36 Nonabortion n = 88 Induced Abortion	V1–V3	NS	Roche/454 GS Junior	[73]
n = 161 pregnancies	n = 83 Control n = 64 1st Trimester Miscarriage n = 14 2nd Trimester Miscarriage	V1–V2	Illumina i5 adapter 5′-AATGATACGGCGACCACCGAGATCTACAC-3′ 8–bp bar code 5′-TATGGTAATT-3′ 28F 5′-GAGTTTGATCNTGGCTCAG-3′ Illumina i7 adapter 5′-CAAGCAGAAGACGGCATACGAGAT-3′ 8-bp bar code 5′-AGTCAGTCAG-3′ 388R 5′-TGCTGCCTCCCGTAGGAGT-3′	MiSeq	[18]
n = 164 patients	n = 48 SA n = 116 NP	V3–V4	343F 5′TACGGRAGGCAGCAG-3 798R 5-AGGGTATCTAATCCT3′	MiSeq	[74]
n = 167 patients	n = 74 Control n = 39 Aneuploid miscarriage n = 54 Euploid miscarriage	V1–V2	28F-YM GAGTTTGATYMTGGCTCAG 28F-Borrellia GAGTTTGATCCTGGCTTAG 28F-Chloroflex GAATTTGATCTTGGTTCAG 28F-Bifdo GGGTTCGATTCTGGCTCAG 388R TGCTGCCTCCCGTAGGAGT	MiSeq	[75]
n = 243 patients	n = 243 RM	NS	NS	NS	[76]

NS—not specified. RRF—repeated reproductive failure. RM—recurrent miscarriage. RSA—recurrent spontaneous abortion. RIF—recurrent implantation failure. RA—repetitive abortion. INF—infertility of unknown origin. RPL—recurrent pregnancy loss. bp—base pair. NP—normal pregnancy.

## Data Availability

The datasets used and analyzed during the current study are available from the corresponding author on reasonable request.

## References

[1] Turnbaugh P.J., Ley R.E., Hamady M., Fraser-Liggett C.M., Knight R., Gordon J.I. (2007). The human microbiome project. Nature.

[2] The Integrative HMP (iHMP) Research Network Consortium (2019). The Integrative Human Microbiome Project. Nature.

[3] Peterson J., Garges S., Giovanni M., McInnes P., Wang L., Schloss J.A., Bonazzi V., McEwen J.E., Wetterstrand K.A., Deal C. (2009). The NIH Human Microbiome Project. Genome Res..

[4] Martin D.H. (2012). The Microbiota of the Vagina and Its Influence on Women’s Health and Disease. Am. J. Med. Sci..

[5] Belkaid Y., Hand T.W. (2014). Role of the microbiota in immunity and inflammation. Cell.

[6] Clemente J.C., Ursell L.K., Parfrey L.W., Knight R. (2012). The impact of the gut microbiota on human health: An integrative view. Cell.

[7] van de Wijgert J.H.H.M., Borgdorff H., Verhelst R., Crucitti T., Francis S., Verstraelen H., Jespers V. (2014). The Vaginal Microbiota: What Have We Learned after a Decade of Molecular Characterization?. PLoS ONE.

[8] Cho I., Blaser M.J. (2012). The human microbiome: At the interface of health and disease. Nat. Rev. Genet..

[9] White B.A., Creedon D.J., Nelson K.E., Wilson B.A. (2011). The vaginal microbiome in health and disease. Trends Endocrinol. Metab..

[10] Martin D.H., Marrazzo J.M. (2016). The Vaginal Microbiome: Current Understanding and Future Directions. J. Infect. Dis..

[11] Theis K.R., Florova V., Romero R., Borisov A.B., Winters A.D., Galaz J., Gomez-Lopez N. (2021). Sneathia: An emerging pathogen in female reproductive disease and adverse perinatal outcomes. Crit. Rev. Microbiol..

[12] So K.A., Yang E.J., Kim N.R., Hong S.R., Lee J.-H., Hwang C.-S., Shim S.-H., Lee S.J., Kim T.J. (2020). Changes of vaginal microbiota during cervical carcinogenesis in women with human papillomavirus infection. PLoS ONE.

[13] Ravel J., Moreno I., Simón C. (2021). Bacterial vaginosis and its association with infertility, endometritis, and pelvic inflammatory disease. Am. J. Obstet. Gynecol..

[14] Kindinger L.M., MacIntyre D.A., Lee Y.S., Marchesi J.R., Smith A., McDonald J.A.K., Terzidou V., Cook J.R., Lees C., Israfil-Bayli F. (2016). Relationship between vaginal microbial dysbiosis, inflammation, and pregnancy outcomes in cervical cerclage. Sci. Transl. Med..

[15] Gajer P., Brotman R.M., Bai G., Sakamoto J., Schütte U.M.E., Zhong X., Koenig S.S.K., Fu L., Ma Z.S., Zhou X. (2012). Temporal dynamics of the human vaginal microbiota. Sci. Transl. Med..

[16] Ravel J., Gajer P., Abdo Z., Schneider G.M., Koenig S.S.K., McCulle S.L., Karlebach S., Gorle R., Russell J., Tacket C.O. (2011). Vaginal microbiome of reproductive-age women. Proc. Natl. Acad. Sci. USA.

[17] Drell T., Lillsaar T., Tummeleht L., Simm J., Aaspõllu A., Väin E., Saarma I., Salumets A., Donders G.G.G., Metsis M. (2013). Characterization of the Vaginal Micro- and Mycobiome in Asymptomatic Reproductive-Age Estonian Women. PLoS ONE.

[18] Al-Memar M., Bobdiwala S., Fourie H., Mannino R., Lee Y.S., Smith A., Marchesi J.R., Timmerman D., Bourne T., Bennett P.R. (2020). The association between vaginal bacterial composition and miscarriage: A nested case–control study. BJOG Int. J. Obstet. Gynaecol..

[19] Robinson C.K., Brotman R.M., Ravel J. (2016). Intricacies of assessing the human microbiome in epidemiologic studies. Ann. Epidemiol..

[20] LeCun Y., Bengio Y., Hinton G. (2015). Deep learning. Nature.

[21] Nikoopour E., Singh B. (2014). Reciprocity in microbiome and immune system interactions and its implications in disease and health. Inflamm. Allergy Drug Targets.

[22] Younes J.A., Lievens E., Hummelen R., van der Westen R., Reid G., Petrova M.I. (2018). Women and Their Microbes: The Unexpected Friendship. Trends Microbiol..

[23] Chow J., Mazmanian S.K. (2010). A pathobiont of the microbiota balances host colonization and intestinal inflammation. Cell Host Microbe.

[24] Franasiak J.M., Scott R.T.J. (2015). Reproductive tract microbiome in assisted reproductive technologies. Fertil. Steril..

[25] Tsonis O., Gkrozou F., Paschopoulos M. (2021). Microbiome affecting reproductive outcome in ARTs. J. Gynecol. Obstet. Hum. Reprod..

[26] Muzii L., Di Tucci C., Galati G., Mattei G., Pietrangeli D., Di Donato V., Perniola G., Palaia I., Benedetti Panici P. (2022). The role of microbiota in female fertility and infertility. Minerva Obstet. Gynecol..

[27] Chen C., Song X., Wei W., Zhong H., Dai J., Lan Z., Li F., Yu X., Feng Q., Wang Z. (2017). The microbiota continuum along the female reproductive tract and its relation to uterine-related diseases. Nat. Commun..

[28] Mitchell C.M., Haick A., Nkwopara E., Garcia R., Rendi M., Agnew K., Fredricks D.N., Eschenbach D. (2015). Colonization of the upper genital tract by vaginal bacterial species in nonpregnant women. Am. J. Obstet. Gynecol..

[29] Moreno I., Codoñer F.M., Vilella F., Valbuena D., Martinez-Blanch J.F., Jimenez-Almazán J., Alonso R., Alamá P., Remohí J., Pellicer A. (2016). Evidence that the endometrial microbiota has an effect on implantation success or failure. Am. J. Obstet. Gynecol..

[30] Franasiak J.M., Scott R.T.J. (2015). Introduction: Microbiome in human reproduction. Fertil. Steril..

[31] Moreno I., Franasiak J.M. (2017). Endometrial microbiota-new player in town. Fertil. Steril..

[32] Toson B., Simon C., Moreno I. (2022). The Endometrial Microbiome and Its Impact on Human Conception. Int. J. Mol. Sci..

[33] Greenbaum S., Greenbaum G., Moran-Gilad J., Weintraub A.Y. (2019). Ecological dynamics of the vaginal microbiome in relation to health and disease. Am. J. Obstet. Gynecol..

[34] France M., Alizadeh M., Brown S., Ma B., Ravel J. (2022). Towards a deeper understanding of the vaginal microbiota. Nat. Microbiol..

[35] Souza S.V., Monteiro P.B., Moura G.A., Santos N.O., Fontanezi C.T.B., Gomes I.A., Teixeira C.A. (2023). Vaginal microbioma and the presence of *Lactobacillus* spp. as interferences in female fertility: A review system. JBRA Assist. Reprod..

[36] Cocomazzi G., De Stefani S., Del Pup L., Palini S., Buccheri M., Primiterra M., Sciannamè N., Faioli R., Maglione A., Baldini G.M. (2023). The Impact of the Female Genital Microbiota on the Outcome of Assisted Reproduction Treatments. Microorganisms.

[37] Lehtoranta L., Ala-Jaakkola R., Laitila A., Maukonen J. (2022). Healthy Vaginal Microbiota and Influence of Probiotics Across the Female Life Span. Front. Microbiol..

[38] Shen L., Zhang W., Yuan Y., Zhu W., Shang A. (2022). Vaginal microecological characteristics of women in different physiological and pathological period. Front. Cell. Infect. Microbiol..

[39] Chopra C., Bhushan I., Mehta M., Koushal T., Gupta A., Sharma S., Kumar M., Al Khodor S., Sharma S. (2022). Vaginal microbiome: Considerations for reproductive health. Future Microbiol..

[40] Kwon M.S., Lee H.K. (2022). Host and Microbiome Interplay Shapes the Vaginal Microenvironment. Front. Immunol..

[41] Gupta V.K., Paul S., Dutta C. (2017). Geography, Ethnicity or Subsistence-Specific Variations in Human Microbiome Composition and Diversity. Front. Microbiol..

[42] Romero R., Espinoza J., Mazor M. (2004). Can endometrial infection/inflammation explain implantation failure, spontaneous abortion, and preterm birth after in vitro fertilization?. Fertil. Steril..

[43] Freitas A.C., Chaban B., Bocking A., Rocco M., Yang S., Hill J.E., Money D.M., Hemmingsen S., Reid G., Dumonceaux T. (2017). The vaginal microbiome of pregnant women is less rich and diverse, with lower prevalence of Mollicutes, compared to non-pregnant women. Sci. Rep..

[44] MacIntyre D.A., Chandiramani M., Lee Y.S., Kindinger L., Smith A., Angelopoulos N., Lehne B., Arulkumaran S., Brown R., Teoh T.G. (2015). The vaginal microbiome during pregnancy and the postpartum period in a European population. Sci. Rep..

[45] Aagaard K., Riehle K., Ma J., Segata N., Mistretta T.-A., Coarfa C., Raza S., Rosenbaum S., Van den Veyver I., Milosavljevic A. (2012). A Metagenomic Approach to Characterization of the Vaginal Microbiome Signature in Pregnancy. PLoS ONE.

[46] Spear G.T., French A.L., Gilbert D., Zariffard M.R., Mirmonsef P., Sullivan T.H., Spear W.W., Landay A., Micci S., Lee B.-H. (2014). Human α-amylase Present in Lower-Genital-Tract Mucosal Fluid Processes Glycogen to Support Vaginal Colonization by Lactobacillus. J. Infect. Dis..

[47] Bradford L.L., Ravel J. (2017). The vaginal mycobiome: A contemporary perspective on fungi in women’s health and diseases. Virulence.

[48] Fox C., Eichelberger K. (2015). Maternal microbiome and pregnancy outcomes. Fertil. Steril..

[49] Moosa Y., Kwon D., de Oliveira T., Wong E.B. (2020). Determinants of Vaginal Microbiota Composition. Front. Cell. Infect. Microbiol..

[50] García-Velasco J.A., Budding D., Campe H., Malfertheiner S.F., Hamamah S., Santjohanser C., Schuppe-Koistinen I., Nielsen H.S., Vieira-Silva S., Laven J. (2020). The reproductive microbiome—Clinical practice recommendations for fertility specialists. Reprod. Biomed. Online.

[51] Heil B.A., Paccamonti D.L., Sones J.L. (2019). Role for the mammalian female reproductive tract microbiome in pregnancy outcomes. Physiol. Genom..

[52] Green B.N., Johnson C.D., Adams A. (2006). Writing narrative literature reviews for peer-reviewed journals: Secrets of the trade. J. Chiropr. Med..

[53] Garcia-Grau I., Perez-Villaroya D., Bau D., Gonzalez-Monfort M., Vilella F., Moreno I., Simon C. (2019). Taxonomical and Functional Assessment of the Endometrial Microbiota in A Context of Recurrent Reproductive Failure: A Case Report. Pathogens.

[54] Liu Y., Wong K.K.-W., Ko E.Y.-L., Chen X., Huang J., Tsui S.K.-W., Li T.C., Chim S.S.-C. (2018). Systematic Comparison of Bacterial Colonization of Endometrial Tissue and Fluid Samples in Recurrent Miscarriage Patients: Implications for Future Endometrial Microbiome Studies. Clin. Chem..

[55] Zhang F., Zhang T., Ma Y., Huang Z., He Y., Pan H., Fang M., Ding H. (2019). Alteration of vaginal microbiota in patients with unexplained recurrent miscarriage. Exp. Ther. Med..

[56] Jiao X., Zhang L., Du D., Wang L., Song Q., Liu S. (2022). Alteration of vaginal microbiota in patients with recurrent miscarriage. J. Obstet. Gynaecol..

[57] Fen-Ting L., Shuo Y., Zi Y., Ping Z., Tianliu P., Jingwen Y., Zhenhong Y., Hongying S., Yang Y., Rong L. (2022). An Altered Microbiota in the Lower and Upper Female Reproductive Tract of Women with Recurrent Spontaneous Abortion. Microbiol. Spectr..

[58] Vomstein K., Reider S., Böttcher B., Watschinger C., Kyvelidou C., Tilg H., Moschen A.R., Toth B. (2022). Uterine microbiota plasticity during the menstrual cycle: Differences between healthy controls and patients with recurrent miscarriage or implantation failure. J. Reprod. Immunol..

[59] Soyer Caliskan C., Yurtcu N., Celik S., Sezer O., Kilic S.S., Cetin A. (2022). Derangements of vaginal and cervical canal microbiota determined with real-time PCR in women with recurrent miscarriages. J. Obstet. Gynaecol..

[60] Xu L., Huang L., Lian C., Xue H., Lu Y., Chen X., Xia Y. (2020). Vaginal Microbiota Diversity of Patients with Embryonic Miscarriage by Using 16S rDNA High-Throughput Sequencing. Int. J. Genom..

[61] Liu X., Cao Y., Xie X., Qin X., He X., Shi C., Zeng W., Guo Y., Lin Y. (2021). Association between vaginal microbiota and risk of early pregnancy miscarriage. Comp. Immunol. Microbiol. Infect. Dis..

[62] Fernández L., Castro I., Arroyo R., Alba C., Beltrán D., Rodríguez J.M. (2021). Application of *Ligilactobacillus salivarius* CECT5713 to Achieve Term Pregnancies in Women with Repetitive Abortion or Infertility of Unknown Origin by Microbiological and Immunological Modulation of the Vaginal Ecosystem. Nutrients.

[63] Fan T., Zhong X.-M., Wei X.-C., Miao Z.-L., Luo S.-Y., Cheng H., Xiao Q. (2020). The alteration and potential relationship of vaginal microbiota and chemokines for unexplained recurrent spontaneous abortion. Medicine.

[64] Kyono K., Hashimoto T., Nagai Y., Sakuraba Y. (2018). Analysis of endometrial microbiota by 16S ribosomal RNA gene sequencing among infertile patients: A single-center pilot study. Reprod. Med. Biol..

[65] Shi Y., Yamada H., Sasagawa Y., Tanimura K., Deguchi M. (2022). Uterine endometrium microbiota and pregnancy outcome in women with recurrent pregnancy loss. J. Reprod. Immunol..

[66] Virtanen S., Saqib S., Kanerva T., Nieminen P., Kalliala I., Salonen A. (2021). Metagenome-validated Parallel Amplicon Sequencing and Text Mining-based Annotations for Simultaneous Profiling of Bacteria and Fungi: Vaginal Microbiome and Mycobiota in Healthy Women. https://assets.researchsquare.com/files/rs-321778/v1/6c64918d-8904-429f-81da-c72b17a8e7e4.pdf?c=1631879415.

[67] Peuranpää P., Holster T., Saqib S., Kalliala I., Tiitinen A., Salonen A., Hautamäki H. (2022). Female reproductive tract microbiota and recurrent pregnancy loss: A nested case-control study. Reprod. Biomed. Online.

[68] Guang Y., Shen X., Tan Y., Tang S., Chen J., Zhang L., Wang B., Ye S., Chen X., Yang C. (2022). Systematic analysis of microbiota in pregnant Chinese women and its association with miscarriage. Ann. Transl. Med..

[69] Sun D., Zhao X., Pan Q., Li F., Gao B., Zhang A., Huang H., Xu D., Cheng C. (2022). The association between vaginal microbiota disorders and early missed abortion: A prospective study. Acta Obstet. Gynecol. Scand..

[70] Mori R., Hayakawa T., Hirayama M., Ozawa F., Yoshihara H., Goto S., Kitaori T., Ozaki Y., Sugiura-Ogasawara M. (2023). Cervicovaginal microbiome in patients with recurrent pregnancy loss. J. Reprod. Immunol..

[71] Teixeira Oliveira C.N., Oliveira M.T.S., Martins Oliveira H.B., Coelho Silva L.S., Santos Júnior M.N., Almeida C.F., Amorim A.T., Oliveira M.V., Timenetsky J., Campos G.B. (2021). Ureaplasma parvum alters the immune tolerogenic state in placental tissue and could cause miscarriage. Fertil. Steril..

[72] Zhao F., Chen Y., Gao J., Wu M., Li C., Wang Z., Huang N., Cui L., Du M., Ying C. (2021). Characterization of Vaginal Microbiota in Women with Recurrent Spontaneous Abortion That Can Be Modified by Drug Treatment. Front. Cell. Infect. Microbiol..

[73] Seo S.S., Arokiyaraj S., Kim M.K., Oh H.Y., Kwon M., Kong J.S., Shin M.K., Yu Y.L., Lee J.K. (2017). High Prevalence of *Leptotrichia amnionii*, *Atopobium vaginae*, *Sneathia sanguinegens*, and Factor 1 Microbes and Association of Spontaneous Abortion among Korean Women. Biomed Res. Int..

[74] Chen S., Xue X., Zhang Y., Zhang H., Huang X., Chen X., Deng G., Luo S., Gao J. (2022). Vaginal Atopobium is Associated with Spontaneous Abortion in the First Trimester: A Prospective Cohort Study in China. Microbiol. Spectr..

[75] Grewal K., Lee Y.S., Smith A., Brosens J.J., Bourne T., Al-Memar M., Kundu S., MacIntyre D.A., Bennett P.R. (2022). Chromosomally normal miscarriage is associated with vaginal dysbiosis and local inflammation. BMC Med..

[76] Kuon R.J., Togawa R., Vomstein K., Weber M., Goeggl T., Strowitzki T., Markert U.R., Zimmermann S., Daniel V., Dalpke A.H. (2017). Higher prevalence of colonization with Gardnerella vaginalis and gram-negative anaerobes in patients with recurrent miscarriage and elevated peripheral natural killer cells. J. Reprod. Immunol..

[77] Pelzer E.S., Willner D., Buttini M., Huygens F. (2018). A role for the endometrial microbiome in dysfunctional menstrual bleeding. Antonie Van Leeuwenhoek.

[78] Kadogami D., Nakaoka Y., Morimoto Y. (2020). Use of a vaginal probiotic suppository and antibiotics to influence the composition of the endometrial microbiota. Reprod. Biol..

[79] Craciunas L., Gallos I., Chu J., Bourne T., Quenby S., Brosens J.J., Coomarasamy A. (2019). Conventional and modern markers of endometrial receptivity: A systematic review and meta-analysis. Hum. Reprod. Update.

[80] Onogi S., Ezoe K., Nishihara S., Fukuda J., Kobayashi T., Kato K. (2020). Endometrial thickness on the day of the LH surge: An effective predictor of pregnancy outcomes after modified natural cycle-frozen blastocyst transfer. Hum. Reprod. Open.

[81] Nasioudis D., Forney L.J., Schneider G.M., Gliniewicz K., France M., Boester A., Sawai M., Scholl J., Witkin S.S. (2017). Influence of Pregnancy History on the Vaginal Microbiome of Pregnant Women in their First Trimester. Sci. Rep..

[82] Carosso A., Revelli A., Gennarelli G., Canosa S., Cosma S., Borella F., Tancredi A., Paschero C., Boatti L., Zanotto E. (2020). Controlled ovarian stimulation and progesterone supplementation affect vaginal and endometrial microbiota in IVF cycles: A pilot study. J. Assist. Reprod. Genet..

[83] Verstraelen H., Vilchez-Vargas R., Desimpel F., Jauregui R., Vankeirsbilck N., Weyers S., Verhelst R., De Sutter P., Pieper D.H., Van De Wiele T. (2016). Characterisation of the human uterine microbiome in non-pregnant women through deep sequencing of the V1-2 region of the 16S rRNA gene. PeerJ.

[84] Hashimoto T., Kyono K. (2019). Does dysbiotic endometrium affect blastocyst implantation in IVF patients?. J. Assist. Reprod. Genet..

[85] Franasiak J.M., Werner M.D., Juneau C.R., Tao X., Landis J., Zhan Y., Treff N.R., Scott R.T. (2016). Endometrial microbiome at the time of embryo transfer: Next-generation sequencing of the 16S ribosomal subunit. J. Assist. Reprod. Genet..

[86] Shin N.-R., Whon T.W., Bae J.-W. (2015). Proteobacteria: Microbial signature of dysbiosis in gut microbiota. Trends Biotechnol..

[87] Liu Y., Ko E.Y.-L., Wong K.K.-W., Chen X., Cheung W.-C., Law T.S.-M., Chung J.P.-W., Tsui S.K.-W., Li T.-C., Chim S.S.-C. (2019). Endometrial microbiota in infertile women with and without chronic endometritis as diagnosed using a quantitative and reference range-based method. Fertil. Steril..

[88] Kitaya K., Matsubayashi H., Yamaguchi K., Nishiyama R., Takaya Y., Ishikawa T., Yasuo T., Yamada H. (2016). Chronic Endometritis: Potential Cause of Infertility and Obstetric and Neonatal Complications. Am. J. Reprod. Immunol..

[89] Kitaya K., Matsubayashi H., Takaya Y., Nishiyama R., Yamaguchi K., Takeuchi T., Ishikawa T. (2017). Live birth rate following oral antibiotic treatment for chronic endometritis in infertile women with repeated implantation failure. Am. J. Reprod. Immunol..

[90] Cicinelli E., Trojano G., Mastromauro M., Vimercati A., Marinaccio M., Mitola P.C., Resta L., de Ziegler D. (2017). Higher prevalence of chronic endometritis in women with endometriosis: A possible etiopathogenetic link. Fertil. Steril..

[91] Cicinelli E., Matteo M., Trojano G., Mitola P.C., Tinelli R., Vitagliano A., Crupano F.M., Lepera A., Miragliotta G., Resta L. (2018). Chronic endometritis in patients with unexplained infertility: Prevalence and effects of antibiotic treatment on spontaneous conception. Am. J. Reprod. Immunol..

[92] Aagaard K., Ma J., Antony K.M., Ganu R., Petrosino J., Versalovic J. (2014). The placenta harbors a unique microbiome. Sci. Transl. Med..

[93] Mei C., Yang W., Wei X., Wu K., Huang D. (2019). The Unique Microbiome and Innate Immunity During Pregnancy. Front. Immunol..

[94] Song S.D., Acharya K.D., Zhu J.E., Deveney C.M., Walther-Antonio M.R.S., Tetel M.J., Chia N. (2020). Daily Vaginal Microbiota Fluctuations Associated with Natural Hormonal Cycle, Contraceptives, Diet, and Exercise. mSphere.

[95] Kaur H., Merchant M., Haque M.M., Mande S.S. (2020). Crosstalk Between Female Gonadal Hormones and Vaginal Microbiota Across Various Phases of Women’s Gynecological Lifecycle. Front. Microbiol..

[96] Fang R.-L., Chen L.-X., Shu W.-S., Yao S.-Z., Wang S.-W., Chen Y.-Q. (2016). Barcoded sequencing reveals diverse intrauterine microbiomes in patients suffering with endometrial polyps. Am. J. Transl. Res..

[97] Churchill S.J., Moreno I., Simón C., Lathi R. (2018). The uterine microbiome in recurrent pregnancy loss. Fertil. Steril..

[98] Krog M.C., Hugerth L.W., Fransson E., Bashir Z., Nyboe Andersen A., Edfeldt G., Engstrand L., Schuppe-Koistinen I., Nielsen H.S. (2022). The healthy female microbiome across body sites: Effect of hormonal contraceptives and the menstrual cycle. Hum. Reprod..

[99] Wang Y., Zhao Y., Ge Y., Cen J., Liao Y., Xu G. (2022). Reproductive outcomes and reproductive tract microbiota shift in women with moderate-to-severe intrauterine adhesions following 30-day post-hysteroscopic placement of balloon stents or intrauterine contraceptive devices: A randomized controlled trial. EClinicalMedicine.

[100] Liu N.-N., Zhao X., Tan J.-C., Liu S., Li B.-W., Xu W.-X., Peng L., Gu P., Li W., Shapiro R. (2022). Mycobiome Dysbiosis in Women with Intrauterine Adhesions. Microbiol. Spectr..

[101] Amabebe E., Anumba D.O.C. (2018). The Vaginal Microenvironment: The Physiologic Role of Lactobacilli. Front. Med..

[102] Vieco-Saiz N., Belguesmia Y., Raspoet R., Auclair E., Gancel F., Kempf I., Drider D. (2019). Benefits and Inputs from Lactic Acid Bacteria and Their Bacteriocins as Alternatives to Antibiotic Growth Promoters During Food-Animal Production. Front. Microbiol..

[103] Leoni C., Ceci O., Manzari C., Fosso B., Volpicella M., Ferrari A., Fiorella P., Pesole G., Cicinelli E., Ceci L.R. (2019). Human Endometrial Microbiota at Term of Normal Pregnancies. Genes.

[104] Wu Y.-R., Dong Y.-H., Liu C.-J., Tang X.-D., Zhang N.-N., Shen J., Wu Z., Li X.-R., Shao J.-Y. (2023). Microbiological composition of follicular fluid in patients undergoing IVF and its association with infertility. Am. J. Reprod. Immunol..

[105] Matsumoto A., Yamagishi Y., Miyamoto K., Oka K., Takahashi M., Mikamo H. (2018). Characterization of the vaginal microbiota of Japanese women. Anaerobe.

[106] Husain S., Allotey J., Drymoussi Z., Wilks M., Fernandez-Felix B.M., Whiley A., Dodds J., Thangaratinam S., McCourt C., Prosdocimi E.M. (2020). Effects of oral probiotic supplements on vaginal microbiota during pregnancy: A randomised, double-blind, placebo-controlled trial with microbiome analysis. BJOG Int. J. Obstet. Gynaecol..

[107] Hattori Y., Nakanishi T., Ozaki Y., Nozawa K., Sato T., Sugiura-Ogasawara M. (2007). Uterine Cervical Inflammatory Cytokines, Interleukin-6 and -8, as Predictors of Miscarriage in Recurrent Cases. Am. J. Reprod. Immunol..

[108] Coomarasamy A., Devall A.J., Cheed V., Harb H., Middleton L.J., Gallos I.D., Williams H., Eapen A.K., Roberts T., Ogwulu C.C. (2019). A Randomized Trial of Progesterone in Women with Bleeding in Early Pregnancy. N. Engl. J. Med..

[109] Masumoto S., Terao A., Yamamoto Y., Mukai T., Miura T., Shoji T. (2016). Non-absorbable apple procyanidins prevent obesity associated with gut microbial and metabolomic changes. Sci. Rep..

[110] Guan S.-M., Shu L., Fu S.-M., Liu B., Xu X.-L., Wu J.-Z. (2008). Prevotella intermedia induces matrix metalloproteinase-9 expression in human periodontal ligament cells. FEMS Microbiol. Lett..

[111] Guan S.-M., Shu L., Fu S.-M., Liu B., Xu X.-L., Wu J.-Z. (2009). Prevotella intermedia upregulates MMP-1 and MMP-8 expression in human periodontal ligament cells. FEMS Microbiol. Lett..

[112] Borgdorff H., Gautam R., Armstrong S.D., Xia D., Ndayisaba G.F., van Teijlingen N.H., Geijtenbeek T.B.H., Wastling J.M., van de Wijgert J.H.H.M. (2016). Cervicovaginal microbiome dysbiosis is associated with proteome changes related to alterations of the cervicovaginal mucosal barrier. Mucosal Immunol..

[113] Mozos I. (2015). Mechanisms linking red blood cell disorders and cardiovascular diseases. Biomed Res. Int..

[114] Weiss G. (2009). Iron metabolism in the anemia of chronic disease. Biochim. Biophys. Acta.

[115] Nakata K., Yamasaki M., Iwata T., Suzuki K., Nakane A., Nakamura H. (2000). Anaerobic bacterial extracts influence production of matrix metalloproteinases and their inhibitors by human dental pulp cells. J. Endod..

[116] Melnick A.P., Pereira N., Murphy E.M., Rosenwaks Z., Spandorfer S.D. (2016). How low is too low? Cycle day 28 estradiol levels and pregnancy outcomes. Fertil. Steril..

[117] Arck P.C., Rücke M., Rose M., Szekeres-Bartho J., Douglas A.J., Pritsch M., Blois S.M., Pincus M.K., Bärenstrauch N., Dudenhausen J.W. (2008). Early risk factors for miscarriage: A prospective cohort study in pregnant women. Reprod. Biomed. Online.

[118] Liu Y., Chen H., Feng L., Zhang J. (2021). Interactions between gut microbiota and metabolites modulate cytokine network imbalances in women with unexplained miscarriage. NPJ Biofilms Microbiomes.

[119] Yang H., Guo R., Li S., Liang F., Tian C., Zhao X., Long Y., Liu F., Jiang M., Zhang Y. (2020). Systematic analysis of gut microbiota in pregnant women and its correlations with individual heterogeneity. NPJ Biofilms Microbiomes.

[120] Moreno I., Garcia-Grau I., Perez-Villaroya D., Gonzalez-Monfort M., Bahçeci M., Barrionuevo M.J., Taguchi S., Puente E., Dimattina M., Lim M.W. (2022). Endometrial microbiota composition is associated with reproductive outcome in infertile patients. Microbiome.

[121] Riganelli L., Iebba V., Piccioni M., Illuminati I., Bonfiglio G., Neroni B., Calvo L., Gagliardi A., Levrero M., Merlino L. (2020). Structural Variations of Vaginal and Endometrial Microbiota: Hints on Female Infertility. Front. Cell. Infect. Microbiol..

[122] Moore D.E., Soules M.R., Klein N.A., Fujimoto V.Y., Agnew K.J., Eschenbach D.A. (2000). Bacteria in the transfer catheter tip influence the live-birth rate after in vitro fertilization. Fertil. Steril..

[123] Selman H., Mariani M., Barnocchi N., Mencacci A., Bistoni F., Arena S., Pizzasegale S., Brusco G.F., Angelini A. (2007). Examination of bacterial contamination at the time of embryo transfer, and its impact on the IVF/pregnancy outcome. J. Assist. Reprod. Genet..

[124] Kindinger L.M., Bennett P.R., Lee Y.S., Marchesi J.R., Smith A., Cacciatore S., Holmes E., Nicholson J.K., Teoh T.G., MacIntyre D.A. (2017). The interaction between vaginal microbiota, cervical length, and vaginal progesterone treatment for preterm birth risk. Microbiome.

[125] Gomez-Lopez N., Romero R., Panaitescu B., Leng Y., Xu Y., Tarca A.L., Faro J., Pacora P., Hassan S.S., Hsu C.-D. (2018). Inflammasome activation during spontaneous preterm labor with intra-amniotic infection or sterile intra-amniotic inflammation. Am. J. Reprod. Immunol..

[126] Tabatabaei N., Eren A.M., Barreiro L.B., Yotova V., Dumaine A., Allard C., Fraser W.D. (2019). Vaginal microbiome in early pregnancy and subsequent risk of spontaneous preterm birth: A case-control study. BJOG Int. J. Obstet. Gynaecol..

[127] Leizer J., Nasioudis D., Forney L.J., Schneider G.M., Gliniewicz K., Boester A., Witkin S.S. (2018). Properties of Epithelial Cells and Vaginal Secretions in Pregnant Women when *Lactobacillus crispatus* or *Lactobacillus iners* Dominate the Vaginal Microbiome. Reprod. Sci..

[128] Nasioudis D., Forney L.J., Schneider G.M., Gliniewicz K., France M.T., Boester A., Sawai M., Scholl J., Witkin S.S. (2017). The composition of the vaginal microbiome in first trimester pregnant women influences the level of autophagy and stress in vaginal epithelial cells. J. Reprod. Immunol..

[129] Saxtorph M.H., Hallager T., Persson G., Petersen K.B., Eriksen J.O., Larsen L.G., Hviid T.V., Macklon N. (2020). Assessing endometrial receptivity after recurrent implantation failure: A prospective controlled cohort study. Reprod. Biomed. Online.

[130] Koedooder R., Maghdid D.M., Beckers N.G.M., Schoenmakers S., Kok D.J., Laven J.S.E. (2021). Dynamics of the urinary microbiome in pregnancy and the coincidental predictive value of the microbiota for IVF/IVF-ICSI outcome. Reprod. Biomed. Online.

[131] Iwami N., Kawamata M., Ozawa N., Yamamoto T., Watanabe E., Mizuuchi M., Moriwaka O., Kamiya H. (2023). Therapeutic intervention based on gene sequencing analysis of microbial 16S ribosomal RNA of the intrauterine microbiome improves pregnancy outcomes in IVF patients: A prospective cohort study. J. Assist. Reprod. Genet..

[132] Zou Y., Liu X., Chen P., Wang Y., Li W., Huang R. (2023). The endometrial microbiota profile influenced pregnancy outcomes in patients with repeated implantation failure: A retrospective study. J. Reprod. Immunol..

[133] Chen P., Chen P., Guo Y., Fang C., Li T. (2021). Interaction Between Chronic Endometritis Caused Endometrial Microbiota Disorder and Endometrial Immune Environment Change in Recurrent Implantation Failure. Front. Immunol..

[134] Lozano F.M., Bernabeu A., Lledo B., Morales R., Diaz M., Aranda F.I., Llacer J., Bernabeu R. (2021). Characterization of the vaginal and endometrial microbiome in patients with chronic endometritis. Eur. J. Obstet. Gynecol. Reprod. Biol..

[135] Petrova M.I., Reid G., Vaneechoutte M., Lebeer S. (2017). *Lactobacillus iners*: Friend or Foe?. Trends Microbiol..

[136] Campisciano G., Iebba V., Zito G., Luppi S., Martinelli M., Fischer L., De Seta F., Basile G., Ricci G., Comar M. (2020). *Lactobacillus iners* and *gasseri*, *Prevotella bivia* and HPV Belong to the Microbiological Signature Negatively Affecting Human Reproduction. Microorganisms.

[137] Haahr T., Zacho J., Bräuner M., Shathmigha K., Skov Jensen J., Humaidan P. (2019). Reproductive outcome of patients undergoing in vitro fertilisation treatment and diagnosed with bacterial vaginosis or abnormal vaginal microbiota: A systematic PRISMA review and meta-analysis. BJOG Int. J. Obstet. Gynaecol..

[138] Leitich H., Kiss H. (2007). Asymptomatic bacterial vaginosis and intermediate flora as risk factors for adverse pregnancy outcome. Best Pract. Res. Clin. Obstet. Gynaecol..

[139] Wang J., Li Z., Ma X., Du L., Jia Z., Cui X., Yu L., Yang J., Xiao L., Zhang B. (2021). Translocation of vaginal microbiota is involved in impairment and protection of uterine health. Nat. Commun..

[140] Al-Nasiry S., Ambrosino E., Schlaepfer M., Morré S.A., Wieten L., Voncken J.W., Spinelli M., Mueller M., Kramer B.W. (2020). The Interplay Between Reproductive Tract Microbiota and Immunological System in Human Reproduction. Front. Immunol..

[141] Bardos J., Fiorentino D., Longman R.E., Paidas M. (2019). Immunological Role of the Maternal Uterine Microbiome in Pregnancy: Pregnancies Pathologies and Alterated Microbiota. Front. Immunol..

[142] Benner M., Ferwerda G., Joosten I., van der Molen R.G. (2018). How uterine microbiota might be responsible for a receptive, fertile endometrium. Hum. Reprod. Update.

[143] Shiroda M., Manning S.D. (2020). *Lactobacillus strains* vary in their ability to interact with human endometrial stromal cells. PLoS ONE.

[144] Smith S.D., Dunk C.E., Aplin J.D., Harris L.K., Jones R.L. (2009). Evidence for immune cell involvement in decidual spiral arteriole remodeling in early human pregnancy. Am. J. Pathol..

[145] Moffett A., Shreeve N. (2015). First do no harm: Uterine natural killer (NK) cells in assisted reproduction. Hum. Reprod..

[146] Parazzini F., Chatenoud L., Tozzi L., Di Cintio E., Benzi G., Fedele L. (1998). Induced abortion in the first trimester of pregnancy and risk of miscarriage. Br. J. Obstet. Gynaecol..

[147] Conde-Ferráez L., May A.D.A.C., Carrillo-Martínez J.R., Ayora-Talavera G., del Refugio González-Losa M. (2013). Human papillomavirus infection and spontaneous abortion: A case–control study performed in Mexico. Eur. J. Obstet. Gynecol. Reprod. Biol..

[148] Liu Y., You H., Chiriva-Internati M., Korourian S., Lowery C.L., Carey M.J., Smith C.V., Hermonat P.L. (2001). Display of Complete Life Cycle of Human Papillomavirus Type 16 in Cultured Placental Trophoblasts. Virology.

[149] You H., Liu Y., Agrawal N., Prasad C.K., Chiriva-Internati M., Lowery C.L., Kay H.H., Hermonat P.L. (2003). Infection, replication, and cytopathology of human papillomavirus type 31 in trophoblasts. Virology.

[150] Gomez L.M., Ma Y., Ho C., McGrath C.M., Nelson D.B., Parry S. (2008). Placental infection with human papillomavirus is associated with spontaneous preterm delivery. Hum. Reprod..

[151] Clark D.A., Banwatt D., Croy B.A. (1993). Murine Trophoblast Failure and Spontaneous Abortion. Am. J. Reprod. Immunol..

[152] Martín R., Jiménez E., Olivares M., Marín M.L., Fernández L., Xaus J., Rodríguez J.M. (2006). *Lactobacillus salivarius* CECT 5713, a potential probiotic strain isolated from infant feces and breast milk of a mother–child pair. Int. J. Food Microbiol..

[153] Tachedjian G., Aldunate M., Bradshaw C.S., Cone R.A. (2017). The role of lactic acid production by probiotic *Lactobacillus* species in vaginal health. Res. Microbiol..

[154] Aldunate M., Srbinovski D., Hearps A.C., Latham C.F., Ramsland P.A., Gugasyan R., Cone R.A., Tachedjian G. (2015). Antimicrobial and immune modulatory effects of lactic acid and short chain fatty acids produced by vaginal microbiota associated with eubiosis and bacterial vaginosis. Front. Physiol..

[155] O’Hanlon D.E., Come R.A., Moench T.R. (2019). Vaginal pH measured in vivo: Lactobacilli determine pH and lactic acid concentration. BMC Microbiol..

[156] Martín V., Cárdenas N., Ocaña S., Marín M., Arroyo R., Beltrán D., Badiola C., Fernández L., Rodríguez J.M. (2019). Rectal and Vaginal Eradication of *Streptococcus agalactiae* (GBS) in Pregnant Women by Using *Lactobacillus salivarius* CECT 9145, A Target-specific Probiotic Strain. Nutrients.

[157] Boskey E.R., Cone R.A., Whaley K.J., Moench T.R. (2001). Origins of vaginal acidity: High D/L lactate ratio is consistent with bacteria being the primary source. Hum. Reprod..

[158] O’Hanlon D.E., Moench T.R., Cone R.A. (2011). In vaginal fluid, bacteria associated with bacterial vaginosis can be suppressed with lactic acid but not hydrogen peroxide. BMC Infect. Dis..

[159] O’Hanlon D.E., Moench T.R., Cone R.A. (2013). Vaginal pH and microbicidal lactic acid when lactobacilli dominate the microbiota. PLoS ONE.

[160] Ruíz F.O., Gerbaldo G., García M.J., Giordano W., Pascual L., Barberis I.L. (2012). Synergistic effect between two bacteriocin-like inhibitory substances produced by *Lactobacilli Strains* with inhibitory activity for *Streptococcus agalactiae*. Curr. Microbiol..

[161] Aldunate M., Tyssen D., Johnson A., Zakir T., Sonza S., Moench T., Cone R., Tachedjian G. (2013). Vaginal concentrations of lactic acid potently inactivate HIV. J. Antimicrob. Chemother..

[162] Tyssen D., Wang Y.-Y., Hayward J.A., Agius P.A., DeLong K., Aldunate M., Ravel J., Moench T.R., Cone R.A., Tachedjian G. (2018). Anti-HIV-1 Activity of Lactic Acid in Human Cervicovaginal Fluid. mSphere.

[163] Chew S.Y., Cheah Y.K., Seow H.F., Sandai D., Than L.T.L. (2015). In vitro modulation of probiotic bacteria on the biofilm of *Candida glabrata*. Anaerobe.

[164] Matsubara V.H., Wang Y., Bandara H.M.H.N., Mayer M.P.A., Samaranayake L.P. (2016). Probiotic lactobacilli inhibit early stages of *Candida albicans* biofilm development by reducing their growth, cell adhesion, and filamentation. Appl. Microbiol. Biotechnol..

[165] Chew S.Y., Cheah Y.K., Seow H.F., Sandai D., Than L.T.L. (2015). Probiotic *Lactobacillus rhamnosus* GR-1 and *Lactobacillus reuteri* RC-14 exhibit strong antifungal effects against vulvovaginal candidiasis-causing *Candida glabrata* isolates. J. Appl. Microbiol..

[166] Aarti C., Khusro A., Varghese R., Arasu M.V., Agastian P., Al-Dhabi N.A., Ilavenil S., Choi K.C. (2018). In vitro investigation on probiotic, anti-Candida, and antibiofilm properties of *Lactobacillus pentosus* strain LAP1. Arch. Oral Biol..

[167] Hütt P., Lapp E., Štšepetova J., Smidt I., Taelma H., Borovkova N., Oopkaup H., Ahelik A., Rööp T., Hoidmets D. (2016). Characterisation of probiotic properties in human vaginal *lactobacilli* strains. Microb. Ecol. Health Dis..

[168] Cárdenas N., Martín V., Arroyo R., López M., Carrera M., Badiola C., Jiménez E., Rodríguez J.M. (2019). Prevention of Recurrent Acute Otitis Media in Children Through the Use of *Lactobacillus salivarius* PS7, a Target-Specific Probiotic Strain. Nutrients.

[169] Boris S., Suárez J.E., Vázquez F., Barbés C. (1998). Adherence of human vaginal lactobacilli to vaginal epithelial cells and interaction with uropathogens. Infect. Immun..

[170] Bogado Pascottini O., Spricigo J.F.W., Van Schyndel S.J., Mion B., Rousseau J., Weese J.S., LeBlanc S.J. (2021). Effects of parity, blood progesterone, and non-steroidal anti-inflammatory treatment on the dynamics of the uterine microbiota of healthy postpartum dairy cows. PLoS ONE.

[171] Gärtner M.A., Peter S., Jung M., Drillich M., Einspanier R., Gabler C. (2016). Increased mRNA expression of selected pro-inflammatory factors in inflamed bovine endometrium in vivo as well as in endometrial epithelial cells exposed to *Bacillus pumilus* in vitro. Reprod. Fertil. Dev..

[172] Huttenhower C., Gevers D., Knight R., Abubucker S., Badger J.H., Chinwalla A.T., Creasy H.H., Earl A.M., Fitz Gerald M.G., Fulton R.S. (2012). Structure, function and diversity of the healthy human microbiome. Nature.

[173] O’Hanlon D.E., Lanier B.R., Moench T.R., Cone R.A. (2010). Cervicovaginal fluid and semen block the microbicidal activity of hydrogen peroxide produced by vaginal lactobacilli. BMC Infect. Dis..

[174] Macklaim J.M., Clemente J.C., Knight R., Gloor G.B., Reid G. (2015). Changes in vaginal microbiota following antimicrobial and probiotic therapy. Microb. Ecol. Health Dis..

[175] Martín R., Soberón N., Vaneechoutte M., Flórez A.B., Vázquez F., Suárez J.E. (2008). Characterization of indigenous vaginal lactobacilli from healthy women as probiotic candidates. Int. Microbiol. Off. J. Spanish Soc. Microbiol..

[176] Mendes-Soares H., Suzuki H., Hickey R.J., Forney L.J. (2014). Comparative functional genomics of *Lactobacillus* spp. reveals possible mechanisms for specialization of vaginal lactobacilli to their environment. J. Bacteriol..

[177] France M.T., Mendes-Soares H., Forney L.J. (2016). Genomic Comparisons of *Lactobacillus crispatus* and *Lactobacillus iners* Reveal Potential Ecological Drivers of Community Composition in the Vagina. Appl. Environ. Microbiol..

[178] Macklaim J.M., Gloor G.B., Anukam K.C., Cribby S., Reid G. (2011). At the crossroads of vaginal health and disease, the genome sequence of *Lactobacillus iners* AB-1. Proc. Natl. Acad. Sci. USA.

[179] Vaneechoutte M. (2017). *Lactobacillus iners*, the unusual suspect. Res. Microbiol..

[180] Borgdorff H., Armstrong S.D., Tytgat H.L.P., Xia D., Ndayisaba G.F., Wastling J.M., van de Wijgert J.H.H.M. (2016). Unique Insights in the Cervicovaginal *Lactobacillus iners* and *L. crispatus* Proteomes and Their Associations with Microbiota Dysbiosis. PLoS ONE.

[181] Petricevic L., Domig K.J., Nierscher F.J., Sandhofer M.J., Fidesser M., Krondorfer I., Husslein P., Kneifel W., Kiss H. (2014). Characterisation of the vaginal *Lactobacillus microbiota* associated with preterm delivery. Sci. Rep..

[182] Lepargneur J.-P. (2016). *Lactobacillus crispatus* as biomarker of the healthy vaginal tract. Ann. Biol. Clin..

[183] Anton L., Sierra L.-J., DeVine A., Barila G., Heiser L., Brown A.G., Elovitz M.A. (2018). Common Cervicovaginal Microbial Supernatants Alter Cervical Epithelial Function: Mechanisms by Which *Lactobacillus crispatus* Contributes to Cervical Health. Front. Microbiol..

[184] Feng Y., Yao Z., Klionsky D.J. (2015). How to control self-digestion: Transcriptional, post-transcriptional, and post-translational regulation of autophagy. Trends Cell Biol..

[185] Srinivasan S., Liu C., Mitchell C.M., Fiedler T.L., Thomas K.K., Agnew K.J., Marrazzo J.M., Fredricks D.N. (2010). Temporal variability of human vaginal bacteria and relationship with bacterial vaginosis. PLoS ONE.

[186] Zheng N., Guo R., Yao Y., Jin M., Cheng Y., Ling Z. (2019). *Lactobacillus iners* Is Associated with Vaginal Dysbiosis in Healthy Pregnant Women: A Preliminary Study. Biomed Res. Int..

[187] Myziuk L., Romanowski B., Johnson S.C. (2003). BVBlue test for diagnosis of bacterial vaginosis. J. Clin. Microbiol..

[188] Mårdh P.-A., Novikova N., Niklasson O., Bekassy Z., Skude L. (2003). Leukocyte esterase activity in vaginal fluid of pregnant and non-pregnant women with vaginitis/vaginosis and in controls. Infect. Dis. Obstet. Gynecol..

[189] Lee Y.S., Koo K.-H., Kim H.J., Tian S., Kim T.-Y., Maltenfort M.G., Chen A.F. (2017). Synovial Fluid Biomarkers for the Diagnosis of Periprosthetic Joint Infection: A Systematic Review and Meta-Analysis. J. Bone Joint Surg. Am..

[190] Fredricks D.N., Fiedler T.L., Marrazzo J.M. (2005). Molecular Identification of Bacteria Associated with Bacterial Vaginosis. N. Engl. J. Med..

[191] Culhane J.F., Nyirjesy P., McCollum K., Goldenberg R.L., Gelber S.E., Cauci S. (2006). Variation in vaginal immune parameters and microbial hydrolytic enzymes in bacterial vaginosis positive pregnant women with and without *Mobiluncus* species. Am. J. Obstet. Gynecol..

[192] Afolabi B.B., Moses O.E., Oduyebo O.O. (2016). Bacterial Vaginosis and Pregnancy Outcome in Lagos, Nigeria. Open Forum Infect. Dis..

[193] Ahmadi A., Khodabandehloo M., Ramazanzadeh R., Farhadifar F., Nikkhoo B., Soofizade N., Rezaii M. (2014). Association between Ureaplasma urealyticum endocervical infection and spontaneous abortion. Iran. J. Microbiol..

[194] Mercer B.M., Goldenberg R.L., Meis P.J., Moawad A.H., Shellhaas C., Das A., Menard M.K., Caritis S.N., Thurnau G.R., Dombrowski M.P. (2000). The Preterm Prediction Study: Prediction of preterm premature rupture of membranes through clinical findings and ancillary testing. Am. J. Obstet. Gynecol..

[195] Dasari S., Anandan S.K., Rajendra W., Valluru L. (2016). Role of microbial flora in female genital tract: A comprehensive review. Asian Pacific J. Trop. Dis..

[196] Koumans E.H., Markowitz L.E., Hogan V. (2002). Indications for therapy and treatment recommendations for bacterial vaginosis in nonpregnant and pregnant women: A synthesis of data. Clin. Infect. Dis. Off. Publ. Infect. Dis. Soc. Am..

[197] Bradshaw C.S., Morton A.N., Hocking J., Garland S.M., Morris M.B., Moss L.M., Horvath L.B., Kuzevska I., Fairley C.K. (2006). High recurrence rates of bacterial vaginosis over the course of 12 months after oral metronidazole therapy and factors associated with recurrence. J. Infect. Dis..

[198] Bradshaw C.S., Tabrizi S.N., Fairley C.K., Morton A.N., Rudland E., Garland S.M. (2006). The association of Atopobium vaginae and Gardnerella vaginalis with bacterial vaginosis and recurrence after oral metronidazole therapy. J. Infect. Dis..

[199] De Backer E., Verhelst R., Verstraelen H., Claeys G., Verschraegen G., Temmerman M., Vaneechoutte M. (2006). Antibiotic susceptibility of *Atopobium vaginae*. BMC Infect. Dis..

[200] MacPhee R.A., Hummelen R., Bisanz J.E., Miller W.L., Reid G. (2010). Probiotic strategies for the treatment and prevention of bacterial vaginosis. Expert Opin. Pharmacother..

[201] Sfakianoudis K., Simopoulou M., Nikas Y., Rapani A., Nitsos N., Pierouli K., Pappas A., Pantou A., Markomichali C., Koutsilieris M. (2018). Efficient treatment of chronic endometritis through a novel approach of intrauterine antibiotic infusion: A case series. BMC Womens. Health.

[202] Turovskiy Y., Sutyak Noll K., Chikindas M.L. (2011). The aetiology of bacterial vaginosis. J. Appl. Microbiol..

[203] Nugent R.P., Krohn M.A., Hillier S.L. (1991). Reliability of diagnosing bacterial vaginosis is improved by a standardized method of gram stain interpretation. J. Clin. Microbiol..

[204] Amsel R., Totten P.A., Spiegel C.A., Chen K.C.S., Eschenbach D., Holmes K.K. (1983). Nonspecific vaginitis: Diagnostic criteria and microbial and epidemiologic associations. Am. J. Med..

[205] Hay P.E. (2004). Bacterial vaginosis and miscarriage. Curr. Opin. Infect. Dis..

[206] Sebire N.J. (2001). Choriodecidual inflammatory syndrome (CoDIS) is the leading, and under recognised, cause of early preterm delivery and second trimester miscarriage. Med. Hypotheses.

[207] U.S. Preventive Services Task Force (2008). Screening for bacterial vaginosis in pregnancy to prevent preterm delivery: U.S. Preventive Services Task Force recommendation statement. Ann. Intern. Med..

[208] Giakoumelou S., Wheelhouse N., Cuschieri K., Entrican G., Howie S.E.M., Horne A.W. (2016). The role of infection in miscarriage. Hum. Reprod. Update.

[209] Yudin M.H., Money D.M. (2017). No. 211-Screening and Management of Bacterial Vaginosis in Pregnancy. J. Obstet. Gynaecol. Can. JOGC J. Obstet. Gynaecol. Can..

[210] Donders G.G., Van Calsteren K., Bellen G., Reybrouck R., Van den Bosch T., Riphagen I., Van Lierde S. (2009). Predictive value for preterm birth of abnormal vaginal flora, bacterial vaginosis and aerobic vaginitis during the first trimester of pregnancy. BJOG Int. J. Obstet. Gynaecol..

[211] du Fossé N.A., Lashley E.E.L.O., van Beelen E., Meuleman T., le Cessie S., van Lith J.M.M., Eikmans M., van der Hoorn M.L.P. (2021). Identification of distinct seminal plasma cytokine profiles associated with male age and lifestyle characteristics in unexplained recurrent pregnancy loss. J. Reprod. Immunol..

[212] Baqui A.H., Lee A.C.C., Koffi A.K., Khanam R., Mitra D.K., Dasgupta S.K., Uddin J., Ahmed P., Rafiqullah I., Rahman M. (2019). Prevalence of and risk factors for abnormal vaginal flora and its association with adverse pregnancy outcomes in a rural district in north-east Bangladesh. Acta Obstet. Gynecol. Scand..

[213] Bonneton M., Huynh B.-T., Seck A., Bercion R., Sarr F.D., Delarocque-Astagneau E., Vray M. (2021). Bacterial vaginosis and other infections in pregnant women in Senegal. BMC Infect. Dis..

[214] Lambert J.A., John S., Sobel J.D., Akins R.A. (2013). Longitudinal analysis of vaginal microbiome dynamics in women with recurrent bacterial vaginosis: Recognition of the conversion process. PLoS ONE.

[215] Oh H.Y., Seo S.-S., Kong J.-S., Lee J.-K., Kim M.K. (2015). Association between Obesity and Cervical Microflora Dominated by *Lactobacillus iners* in Korean Women. J. Clin. Microbiol..

[216] Smith B.C., McAndrew T., Chen Z., Harari A., Barris D.M., Viswanathan S., Rodriguez A.C., Castle P., Herrero R., Schiffman M. (2012). The cervical microbiome over 7 years and a comparison of methodologies for its characterization. PLoS ONE.

[217] Zhou X., Brown C.J., Abdo Z., Davis C.C., Hansmann M.A., Joyce P., Foster J.A., Forney L.J. (2007). Differences in the composition of vaginal microbial communities found in healthy Caucasian and black women. ISME J..

[218] Bobdiwala S., Al-Memar M., Lee Y., Smith A., Marchesi J., Bennett P., Bourne T., MacIntyre D. (2017). OC08.06: *Association of vaginal microbiota composition and outcomes in women found to have a pregnancy of unknown location (PUL) on an initial early pregnancy ultrasound scan. Ultrasound Obstet. Gynecol..

[219] Mangalam A., Shahi S.K., Luckey D., Karau M., Marietta E., Luo N., Choung R.S., Ju J., Sompallae R., Gibson-Corley K. (2017). Human Gut-Derived Commensal Bacteria Suppress CNS Inflammatory and Demyelinating Disease. Cell Rep..

[220] Liblau R. (2009). Glatiramer acetate for the treatment of multiple sclerosis: Evidence for a dual anti-inflammatory and neuroprotective role. J. Neurol. Sci..

[221] Nelson D.B., Bellamy S., Nachamkin I., Ness R.B., Macones G.A., Allen-Taylor L. (2007). First trimester bacterial vaginosis, individual microorganism levels, and risk of second trimester pregnancy loss among urban women. Fertil. Steril..

[222] Kumar M., Murugesan S., Singh P., Saadaoui M., Elhag D.A., Terranegra A., Kabeer B.S.A., Marr A.K., Kino T., Brummaier T. (2021). Vaginal Microbiota and Cytokine Levels Predict Preterm Delivery in Asian Women. Front. Cell. Infect. Microbiol..

[223] Sharma H., Tal R., Clark N.A., Segars J.H. (2014). Microbiota and pelvic inflammatory disease. Semin. Reprod. Med..

[224] Karaer A., Doğan B., Günal S., Tuncay G., Arda Düz S., Ünver T., Tecellioğlu N. (2021). The vaginal microbiota composition of women undergoing assisted reproduction: A prospective cohort study. BJOG Int. J. Obstet. Gynaecol..

[225] Amabebe E., Reynolds S., Stern V.L., Parker J.L., Stafford G.P., Paley M.N., Anumba D.O.C. (2016). Identifying metabolite markers for preterm birth in cervicovaginal fluid by magnetic resonance spectroscopy. Metabolomics.

[226] Rosca A.S., Castro J., Sousa L.G.V., Cerca N. (2020). Gardnerella and vaginal health: The truth is out there. FEMS Microbiol. Rev..

[227] Odogwu N.M., Chen J., Onebunne C.A., Jeraldo P., Yang L., Johnson S., Ayeni F.A., Walther-Antonio M.R.S., Olayemi O.O., Chia N. (2021). Predominance of Atopobium vaginae at Midtrimester: A Potential Indicator of Preterm Birth Risk in a Nigerian Cohort. mSphere.

[228] Fettweis J.M., Serrano M.G., Brooks J.P., Edwards D.J., Girerd P.H., Parikh H.I., Huang B., Arodz T.J., Edupuganti L., Glascock A.L. (2019). The vaginal microbiome and preterm birth. Nat. Med..

[229] Hočevar K., Maver A., Vidmar Šimic M., Hodžić A., Haslberger A., Premru Seršen T., Peterlin B. (2019). Vaginal Microbiome Signature Is Associated with Spontaneous Preterm Delivery. Front. Med..

[230] Larsen B., Hwang J. (2010). Mycoplasma, Ureaplasma, and adverse pregnancy outcomes: A fresh look. Infect. Dis. Obstet. Gynecol..

[231] Pace R.M., Chu D.M., Prince A.L., Ma J., Seferovic M.D., Aagaard K.M. (2021). Complex species and strain ecology of the vaginal microbiome from pregnancy to postpartum and association with preterm birth. Med.

[232] Romero R., Hassan S.S., Gajer P., Tarca A.L., Fadrosh D.W., Bieda J., Chaemsaithong P., Miranda J., Chaiworapongsa T., Ravel J. (2014). The vaginal microbiota of pregnant women who subsequently have spontaneous preterm labor and delivery and those with a normal delivery at term. Microbiome.

[233] Elovitz M.A., Gajer P., Riis V., Brown A.G., Humphrys M.S., Holm J.B., Ravel J. (2019). Cervicovaginal microbiota and local immune response modulate the risk of spontaneous preterm delivery. Nat. Commun..

[234] Fang J., Chen L., Chen Z., Jiang X., Pan M. (2020). Association of the vaginal microbiota with pregnancy outcomes in Chinese women after cervical cerclage. Reprod. Biomed. Online.

[235] Brown R.G., Chan D., Terzidou V., Lee Y.S., Smith A., Marchesi J.R., MacIntyre D.A., Bennett P.R. (2019). Prospective observational study of vaginal microbiota pre- and post-rescue cervical cerclage. BJOG Int. J. Obstet. Gynaecol..

[236] Garry N., Keenan O., Lindow S.W., Darcy T. (2021). Pregnancy outcomes following elective abdominal cerclage following cervical excision surgery for neoplastic disease. Eur. J. Obstet. Gynecol. Reprod. Biol..

[237] Vomstein K., Egerup P., Kolte A.M., Behrendt-Møller I., Boje A.D., Bertelsen M.-L., Eiken C.S., Reiersen M.R., Toth B., la Cour Freiesleben N. (2023). Biopsy-free profiling of the uterine immune system in patients with recurrent pregnancy loss and unexplained infertility. Reprod. Biomed. Online.

[238] Miles S.M., Hardy B.L., Merrell D.S. (2017). Investigation of the microbiota of the reproductive tract in women undergoing a total hysterectomy and bilateral salpingo-oopherectomy. Fertil. Steril..

[239] Moreno I., Cicinelli E., Garcia-Grau I., Gonzalez-Monfort M., Bau D., Vilella F., De Ziegler D., Resta L., Valbuena D., Simon C. (2018). The diagnosis of chronic endometritis in infertile asymptomatic women: A comparative study of histology, microbial cultures, hysteroscopy, and molecular microbiology. Am. J. Obstet. Gynecol..

[240] Tao X., Franasiak J.M., Zhan Y., Scott R.T., Rajchel J., Bedard J., Newby R., Scott R.T., Treff N.R., Chu T. (2017). Characterizing the endometrial microbiome by analyzing the ultra-low bacteria from embryo transfer catheter tips in IVF cycles: Next generation sequencing (NGS) analysis of the 16S ribosomal gene. Hum. Microbiome J..

[241] Moreno I., Garcia-Grau I., Bau D., Perez-Villaroya D., Gonzalez-Monfort M., Vilella F., Romero R., Simón C. (2020). The first glimpse of the endometrial microbiota in early pregnancy. Am. J. Obstet. Gynecol..

[242] Chang D.-H., Shin J., Rhee M.-S., Park K.-R., Cho B.-K., Lee S.-K., Kim B.-C. (2020). Vaginal Microbiota Profiles of Native Korean Women and Associations with High-Risk Pregnancy. J. Microbiol. Biotechnol..

[243] Winters A.D., Romero R., Gervasi M.T., Gomez-Lopez N., Tran M.R., Garcia-Flores V., Pacora P., Jung E., Hassan S.S., Hsu C.-D. (2019). Does the endometrial cavity have a molecular microbial signature?. Sci. Rep..

[244] Hyman R.W., Fukushima M., Jiang H., Fung E., Rand L., Johnson B., Vo K.C., Caughey A.B., Hilton J.F., Davis R.W. (2014). Diversity of the vaginal microbiome correlates with preterm birth. Reprod. Sci..

[245] Shi Y., Tanimura K., Sasagawa Y., Yamada H. (2020). Vaginal microbiota associated with preterm delivery. J. Infect. Chemother. Off. J. Japan Soc. Chemother..

[246] Payne M.S., Newnham J.P., Doherty D.A., Furfaro L.L., Pendal N.L., Loh D.E., Keelan J.A. (2021). A specific bacterial DNA signature in the vagina of Australian women in midpregnancy predicts high risk of spontaneous preterm birth (the Predict1000 study). Am. J. Obstet. Gynecol..

[247] Brown R.G., Al-Memar M., Marchesi J.R., Lee Y.S., Smith A., Chan D., Lewis H., Kindinger L., Terzidou V., Bourne T. (2019). Establishment of vaginal microbiota composition in early pregnancy and its association with subsequent preterm prelabor rupture of the fetal membranes. Transl. Res..

[248] Mitchell C.M., Mazzoni C., Hogstrom L., Bryant A., Bergerat A., Cher A., Pochan S., Herman P., Carrigan M., Sharp K. (2020). Delivery Mode Affects Stability of Early Infant Gut Microbiota. Cell Rep. Med..

